# Advancing HIV Broadly Neutralizing Antibodies: From Discovery to the Clinic

**DOI:** 10.3389/fpubh.2021.690017

**Published:** 2021-05-26

**Authors:** David A. Spencer, Mariya B. Shapiro, Nancy L. Haigwood, Ann J. Hessell

**Affiliations:** ^1^Oregon National Primate Research Center, Oregon Health & Science University, Beaverton, OR, United States; ^2^Molecular Microbiology & Immunology Department, School of Medicine, Oregon Health & Science University, Portland, OR, United States

**Keywords:** HIV, antibody, non-human primate, immunotherapy, neutralization

## Abstract

Despite substantial progress in confronting the global HIV-1 epidemic since its inception in the 1980s, better approaches for both treatment and prevention will be necessary to end the epidemic and remain a top public health priority. Antiretroviral therapy (ART) has been effective in extending lives, but at a cost of lifelong adherence to treatment. Broadly neutralizing antibodies (bNAbs) are directed to conserved regions of the HIV-1 envelope glycoprotein trimer (Env) and can block infection if present at the time of viral exposure. The therapeutic application of bNAbs holds great promise, and progress is being made toward their development for widespread clinical use. Compared to the current standard of care of small molecule-based ART, bNAbs offer: (1) reduced toxicity; (2) the advantages of extended half-lives that would bypass daily dosing requirements; and (3) the potential to incorporate a wider immune response through Fc signaling. Recent advances in discovery technology can enable system-wide mining of the immunoglobulin repertoire and will continue to accelerate isolation of next generation potent bNAbs. Passive transfer studies in pre-clinical models and clinical trials have demonstrated the utility of bNAbs in blocking or limiting transmission and achieving viral suppression. These studies have helped to define the window of opportunity for optimal intervention to achieve viral clearance, either using bNAbs alone or in combination with ART. None of these advances with bNAbs would be possible without technological advancements and expanding the cohorts of donor participation. Together these elements fueled the remarkable growth in bNAb development. Here, we review the development of bNAbs as therapies for HIV-1, exploring advances in discovery, insights from animal models and early clinical trials, and innovations to optimize their clinical potential through efforts to extend half-life, maximize the contribution of Fc effector functions, preclude escape through multiepitope targeting, and the potential for sustained delivery.

## Introduction

The HIV/AIDS pandemic remains one of the greatest public health challenges of our time. Since its discovery in the early 1980s, the AIDS-causing virus HIV-1 (HIV) has infected 75 million people worldwide and claimed 32 million lives (1). In 2018, there were 38 million people living with HIV, of which 1.7 million were children under 15 years of age (1). Despite decades of research and a number of clinical trials, a vaccine for HIV remains elusive. While the number of annual deaths due to AIDS and new HIV infections has declined by 33% and 16%, respectively, over the last decade due to the widespread use of effective antiretroviral drug therapy (1), better treatments are urgently needed to address the pandemic more effectively. Today, the management of HIV with cocktails of antiretroviral drugs, or “antiretroviral therapy” (ART), maintains plasma virus at undetectable levels as long as the drug is present at therapeutic levels *in vivo*, but does not clear viral reservoirs, and if discontinued the virus rebounds (2). ART compounds are small molecules that typically have short half-lives, necessitating treatment daily for ongoing protection in adults and children exposed to HIV by breastfeeding. The recent development of improved formulations of ART that require infrequent dosing will ultimately improve adherence (3, 4), but it will not fully mitigate risks due to the necessity for lifelong adherence to drugs that possess significant inherent toxicities, which manifest generally as fatigue and malaise in many ART recipients, and may include myopathy, neuropathy, hepatic failure, and lactic acidosis from ART-induced mitochondrial dysregulation (5, 6). All of these factors work together to result in suboptimal or sporadic treatment, which raises the risk of viral resistance and treatment failure. In addition to therapeutic indications, ART is used by uninfected at-risk people to prevent infection, which is known as pre-exposure prophylaxis (PrEP); this approach is available in the US and several other countries, but not yet worldwide (7). As a less toxic and longer lasting alternative to ART, the use of monoclonal antibodies (mAbs), delivered passively or by gene therapy, is being explored in pre-clinical or clinical trials, but small-molecule ART remains the only FDA-approved treatment modality for HIV/AIDS in 2021.

Passive immunotherapy was originally pioneered at the turn of the century with the use of horse serum to neutralize diphtheria toxin in infected subjects, and the clinical benefit showed the power of antibodies to mitigate disease (8). The discoveries of the intervening 120 years have led to some very tangible and, more recently, game-changing advances in potential applications for HIV. When HIV was discovered to be the causative agent of AIDS, intensive research ensued to develop diagnostic tests and vaccines. Once it became apparent that some subjects progressed more rapidly to disease than others, efforts were directed to characterize anti-HIV adaptive responses and the causes of CD4 depletion. These early studies showed that HIV-specific CD8+ T cell responses, and not antibodies, were coincident with the decline of acute virus production in the blood; subsequent data showed that certain HLA haplotypes were associated with better virus control and longer lives in “elite controllers,” reviewed by Deeks and Walker (9). Antibodies directed to HIV antigens did develop, but the appearance of neutralizing antibodies (NAbs)—directed to Env and capable of neutralizing the virus in tissue culture—took months. Furthermore, higher levels of antibodies and NAbs were seen in subjects with higher levels of plasma viremia and were not associated with diminution of virus in elite controllers (10). These data led to the concept that it is primarily CD8+ T cells that are responsible for viral control and that neutralizing antibodies arose too late to deal with a retroviral infection. Further confounding the field were early experiments to test HIV immune globulin (HIVIG), IgG purified from HIV-positive subjects, as passive immunotherapy, in chimpanzees, the only animal model available at the time. Despite the eventual success of HIVIG in protecting against challenge at high doses (11), as well as several other proof-of-principle studies using polyclonal IgG, the difficulties inherent in developing a polyclonal IgG product for widespread therapeutic administration underscored the need for more targeted approaches using monoclonal antibodies (12–14).

**Figure 1:**
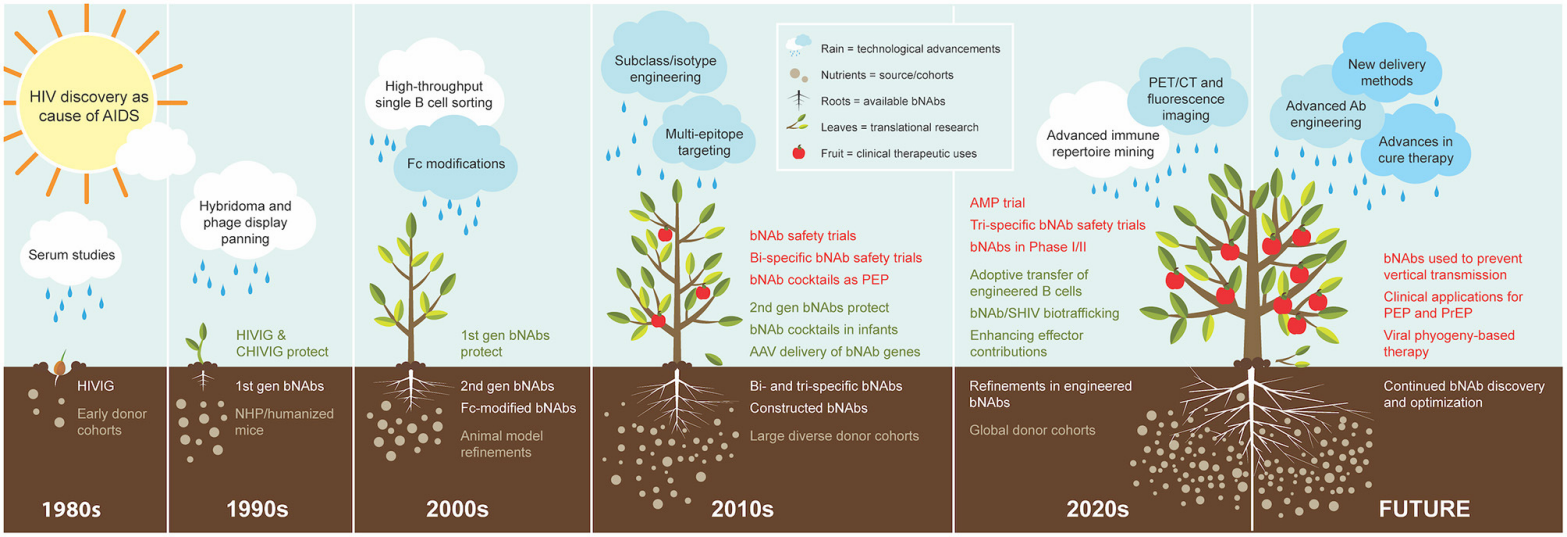
Cultivation of bNAbs for treatment and prevention of HIV. The progression of bNAb development is visualized as organic growth seeded from observations in the 1980s−1990s that passive transfer of virus-neutralizing polyclonal IgG from subjects living with HIV countered new infection. The subsequent yield of 1st generation bNAbs in the 1990s demonstrated protection from SHIV in NHP models, and B cell technology advancement in the 2000s enabled the isolation of the 2nd generation bNAbs that are highly broad and potent and protected humanized mice and NHP models at low doses. The cache of 2nd generation bNAbs quickly expanded in the 2010s as cohorts of large and diverse study subjects also multiplied. The swift advancement of these bNAbs to the clinic in the same decade was nurtured by technological advancements in molecular and cellular biology and systems immunology, and their applications in the clinic continue to increase. The 2020s has begun with the recent completion of the first large-scale human efficacy trial with one of the early 2nd generation bNAbs. Advanced immune repertoire mining technology is becoming a new standard for discovery, and studies are underway with new modification ideas. Expectations for future clinical applications include novel concepts for delivery methods and utilizing engineered bNAb cocktails. The figure legend illustrates and describes each element fostering a continuum in growth of bNAb utility over time. Elements are presented in the decade of first use, although concepts, processes, and materials may continue over multiple decades.

**Figure 2:**
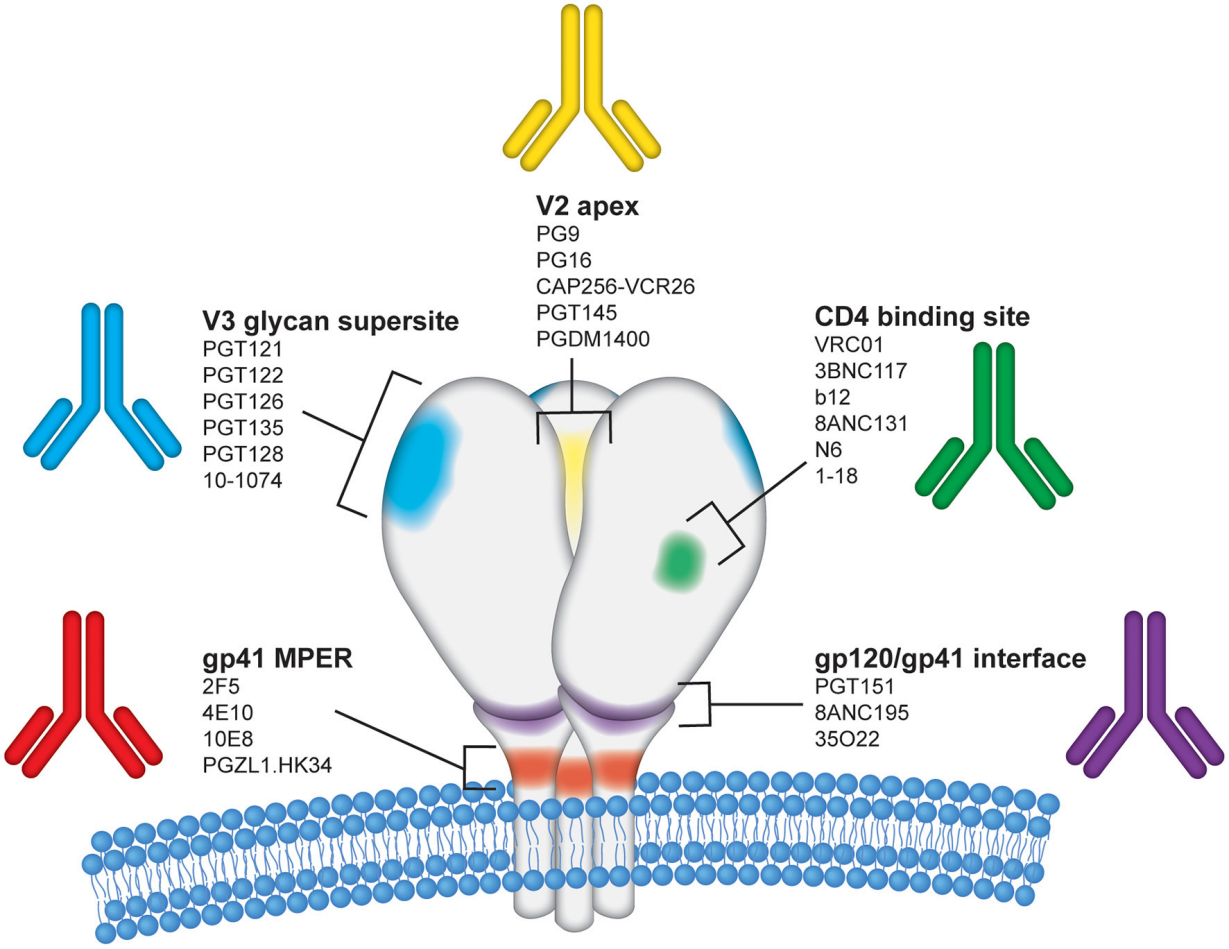
Epitope regions on the HIV-1 Envelope targeted by bNAbs. The HIV-1 envelope glycoprotein (Env) is the sole target for bNAbs and is displayed on the surface of virions as a trimer of gp120 and gp41 heterodimers. Each of the three gp120 protomers are shown in white above the viral membrane with the three gp41 regions shown embedded within the viral membrane. For simplicity, glycans and variable regions on HIV Env are not depicted in this figure. The image is an artistic rendering to illustrate the five primary conserved epitope regions on the HIV Env surface proteins targeted by bNAbs. The epitope regions shown are labeled as follows: (1) V2 apex–yellow; (2) CD4 binding site–green; (3) gp120/gp41 interface–purple; (4) gp41 membrane proximal region (MPER)–red; and (5) V3 glycan supersite–blue. Several representative human monoclonal antibodies known to target each of these regions are listed.

Building from the early work of extracting mAbs from human bone marrow and PBMCs that developed during infection, one of the critical areas of intense exploration in HIV research in the last several decades has been the cloning and characterization of NAbs from infected subjects who develop potent neutralizing plasma Abs against a broad spectrum of HIV circulating strains. Innovative work from studies of human cohorts quickly led to the discovery and cloning of these powerful mAbs to use as therapies (Figure 1). At the same time, HIV mAb research has shed light on mechanisms for antibody epitope targeting and maturation in the face of viral escape variants. The native immune response is typically ineffective at preventing HIV infection in the face of repeated exposures, in part because the virion restricts presentation of non-self epitopes. As an enveloped virus, HIV displays surface proteins composed of a trimer of gp120 and gp41 heterodimers, and their sparse distribution, with an estimated 4–35 trimers per virion, limits the potential for immune receptor crosslinking and downstream activation (15). Immunogenicity is further reduced by extensive trimer glycosylation, which effectively shields them from B cell receptor (BCR) recognition (16–18). Prior to receptor attachment, which induces a more relaxed “state 3” conformation accessible to neutralizing antibodies, Env trimers oscillate between partially relaxed “state 2” and closed “state 1” conformations (19, 20) that present five known highly conserved epitopes susceptible to neutralizing antibodies (Figure 2), reviewed by Kwong and Mascola (21). Despite these defenses, a very small minority of individuals living with HIV develop bNAbs to one or more of these epitopes, but typically do so several years after chronic infection drives extensive BCR somatic hypermutation (22–24). Although their development in response to natural infection is not widespread, these exceptional bNAbs neutralize the majority of strains on multi-clade pseudovirus panels representing the global diversity of circulating HIV, often at very low concentrations with average IC_50_s <1 μg/ml, by blocking a critical Env function such as receptor or co-receptor attachment or membrane fusion (25). Notably, this breadth is achieved due to the highly conserved primary amino acid sequences on Env mediating these functions, with the greatest breadth observed in bNAbs targeting the membrane external proximal region (MPER) or the CD4 binding site (CD4bs) (25). When produced as monoclonals and passively transferred, bNAbs have been shown to be highly effective in preventing disease in animal models, and they offer promising prophylactic and therapeutic tools to restrict transmission and control disease progression. This review surveys the issues and promise of bNAbs as preventive and therapeutic tools for HIV, with representative key examples of success in animal models and in the clinic.

## Cultivating bNAb Development For Treatment And Prevention

### Expanding the Therapeutic bNAb Toolkit

First generation “bNAbs” isolated from screening Abs cloned from Env^+^ memory B cells showed the prophylactic potential of NAbs prior to challenge with HIV in humanized mice (26–28) or challenge with simian immunodeficiency virus expressing HIV Env (SHIV) in NHPs (29–33). The subsequent isolation of more potent bNAbs through high throughput functional screening of single B cells prior to cloning yielded second generation bNAbs capable of protecting NHPs challenged with difficult to neutralize tier 2 SHIV at plasma concentrations as low 1 μg/ml (34–37), and several of these bNAbs are being evaluated in early stage clinical trials (38–40). Improved stabilization of Env trimers that offer more stringent presentation of native like conformational epitopes can now be utilized as sorting probes. When used in multi-clade combinations, these probes favor the selection of B cells targeting the rare but highly conserved bNAb epitopes, and thus permit a more extensive functional characterization of only the most promising putative bNAb candidates. For example, this approach has yielded the recent discovery of novel bNAb 1–18, which displays near pan neutralizing breadth with an average IC_50_ below 0.1 μg/ml against pseudovirus panels designed to represent the global diversity of viral strains, possesses a different germline lineage than the canonical CD4bs VRC01-like bNAb family (V_H_1–46 instead of V_H_1–2), and correspondingly targets the CD4bs in a distinct manner (41). The discovery of bNAb 1–18 holds a particular significance because of its dual capacity for exceptional neutralizing activity combined with an extremely efficient impediment to viral escape. These combined characteristics in a single bNAb will ultimately aid other efforts to maximize bNAb utility by rational cocktail design and engineering bi- and tri-specific molecules as discussed more below.

Once a bNAb is identified from donor plasma, antibodies belonging to the same lineage can be rapidly identified by immunoglobulin sequencing of longitudinal samples, an approach which is enabled by next generation sequencing and showcased by the robust lineage analysis for bNAb VRC34, and provides significant insight into bNAb development and optimal HC/LC pairings (42). Alternative HC/LC pairings can then be sequentially evaluated to determine the most potent combination that may or may not have arisen naturally. An example of the success of this approach is seen in the isolation of 4E10-like MPER-targeting bNAb PGZL1 HC paired with the H4K3 LC. The resulting PGZL1.H4K3 displays pan neutralizing breadth with an average IC_50_ of 1.4 μg/ml against a 130 pseudovirus panel compared to 84% breadth and an average IC_50_ of 6.1 μg/ml of parental PGZL1 (43). The significance of the PGZL1.HK43 discovery has important implications for vaccine development. First, the parental mAb PGZL1 was isolated as an IgG1—not IgG3—with a relatively short CDRH3, little hydrophobicity or polyreactivity unlike most MPER mAbs. Next, unlike that of 4E10 and 10E8, the germline revertant PGZL1.HK43 binds MPER and neutralizes HIV. These characteristics suggest that HK43 and other 4E10-mAbs could potentially be elicited by vaccine immunogens designed more specifically for distal MPER epitopes.

Ontogeny based approaches are increasingly being utilized to both advance bNAb discovery and to inform rational design of vaccine immunogens. An example is work done by Williams et al. with the clonal lineage of DH511 that was isolated in studies of ontogeny of antibodies derived from both memory B cells and plasma of a clade C-infected African donor (44). MPER targeting bnAbs 4E10, 10E8, and DH511 are extremely broad neutralizers of HIV isolates and clades and target nearly identical α-helical epitopes in the MPER (45). The drawback for MPER antibodies has been features of lipid residue interaction and polyreactivity (46, 47). While the proximal MPER bNAb 2F5 does not capture infectious virions, both distal MPER bNAbs, DH511, and 4E10 have this capacity and also bind in similar context to membrane lipids (44). Earlier cryo-EM work with a fully glycosylated, cleaved Env has demonstrated that distal binding MPER mAbs bind to both lipid and the prefusion conformation of Env gp41 before CD4-induced Env activation (48). This study made important observations regarding the dynamic topology at the viral membrane that complicate targeting by the immune system.

More comprehensive and efficient approaches to bNAb identification continue to emerge. Advances in single cell multiplexing now allow for the rapid and almost system-wide screening of the expressed immunoglobulin repertoire paired with functional BCR binding data to an antigen of interest. One such approach, called linking B cell receptor to antigen specificity through sequencing (LIBRA-seq), utilizes oligonucleotides to uniquely barcode each fluorescently labeled sorting probe (49). A mixture of barcoded probes is then incubated with PBMCs or other pooled cell subsets of interest. Antigen positive B cells are sorted by fluorescence, and then partitioned into single cell droplets for next generation sequencing, with an end readout of IgH and IgL sequences paired with antigen binding enabled by the oligonucleotide barcode. Hence, the paired IgH/IgL sequences of B cells cross reactive to multiple conformationally constrained Env trimers can be rapidly ascertained (49). Notably, these advances in monoclonal isolation are broadly applicable beyond HIV and are responsible for an exponentially expanding number of monoclonal antibody candidates for clinical use against other viruses, including for SARS-Cov-2, influenza, Ebola, and others (50–54).

### Insights Into Clinical Indications From Animal Models

Animal models have proved essential for evaluating HIV neutralizing antibody dynamics and efficacy *in vivo*. Studies of wild chimpanzees confirmed the origins of HIV and the presence of disease in that species (55). Chimpanzees were tested early in the epidemic as research models and were shown to be infectable, but disease was rare and only seen after prolonged infection (56). Due to the tight host range restriction of immunodeficiency virus in humans and chimpanzees, it has been necessary to modify the host or the virus to develop more useful models for HIV infection and pathogenesis. Humanized mouse models were developed by using mice deficient in immunity (severe combined immune deficient, or SCID) and engrafted with human peripheral blood mononuclear cells, hematopoietic stem cells, or human bone marrow, liver, and thymus (so called BLT mice) to make them permissive to infection with HIV, reviewed by Shultz and colleagues (57). Several additional models have been developed in recent years to further refine this resource and to increase the ability of the strains to mount human adaptive responses (58). Although humanized mice recapitulate some aspects of HIV reservoir establishment and disease pathogenesis seen in humans, their value is primarily in testing the ability of bNAbs to block infection since they have relatively short lives and do not recapitulate all aspects of adaptive immunity or disease progression in people. Nonetheless, several groups have shown that these mouse models are suitable for testing passive transfer of bNAbs for efficacy at blocking infection, as noted in the previous section (28, 59). Humanized mice also have been used to show the utility of AAV-delivered antibodies expressed *in vivo* to provide resistance to intravenous or mucosal challenge (60).

Over the last 35 years, non-human primates (NHPs) have emerged as an indispensable model for studying HIV pathogenesis, vaccines, and therapies. The fortuitous discovery of pathogenesis following infection of Asian macaques by African simian immunodeficiency viruses (SIV from sooty mangabeys, *Cercocebus atys*) led to NHP models for HIV transmission, pathogenesis, and immunity (61). One drawback initially limiting use of this model for antibody-based studies was that SIV Env is antigenically distinct and is not recognized by HIV bNAbs. To solve this problem, chimeric simian/human immunodeficiency viruses (SHIVs) have been constructed by inserting HIV Env into the SIV (usually SIV_mac239_) backbone (62, 63). The result is a chimeric virus with most SIV proteins intact, but carrying Env from a particular HIV isolate. An additional mutation has been added to several new SHIV strains to improve binding of the Env to the macaque CD4 receptor (64) and further improvements continue to be made (65, 66). The disease course for SIV and SHIV are similar and somewhat variable depending upon the strain, but overall pathogenesis is measured in months to a few years, which is much more rapid than that of HIV in humans (65, 67, 68). Nevertheless, NHP models using SHIV infection have contributed substantially to the advancement of HIV bNAb use in both adult (69, 70) and pediatric settings (71).

The earliest experiments in chimpanzees revealed that polyclonal IgG purified from an infected individual (HIVIG) with neutralizing activity could block HIV infection, after two failed attempts, when used at a sufficiently high dose (11). However, all of the chimpanzee experiments were performed in very small groups to conserve this scarce animal resource. Two pivotal experiments published in 1999 showed that IgG purified from an HIV-infected chimpanzee (termed “CHIVIG”) with potent neutralizing activity could block infection in macaques challenged with SHIV in a dose-dependent manner, while HIVIG purified from uninfected subjects with no HIV neutralization could not protect from challenge (72). Furthermore, CHIVIG was shown to reduce the infectivity of SHIV *in vivo* in macaques (73). Numerous follow-up PrEP studies have shown that passive immunization with bNAbs at much lower doses than HIVIG, due to increased specific activity of these monoclonals, effectively protects macaques from single or repeated SHIV challenge (34, 74–76), summarized in a recent review (70). These studies definitively showed the importance of neutralization in protection, and they have helped to define the levels of neutralization required for protection, as diverse strains of SHIV require different doses of antibody (77). In addition to neutralization, many bNAbs mediate antiviral activities such as antibody dependent cellular cytotoxicity (ADCC) and phagocytosis (ADCP) *via* FcγR engagement, and these functions may contribute to their protective efficacy as discussed more below (78). Nonetheless, protection studies with non-NAbs have further confirmed that neutralizing activity is required for protection (79–81), and the potency of neutralization *in vitro* correlates with protective efficacy *in vivo* (82).

Recent post-exposure prophylaxis (PEP) studies in macaques have shed light on the window of opportunity for effective bNAb therapy after SHIV challenge (Table 1), building on early data showing IgG therapy in SIV-infected macaques using neutralizing “SIVIG” as PEP resulted in viral suppression and protection from disease progression (83). Using a rhesus macaque model of perinatal infection, our group presented the first evidence that early post-exposure prophylaxis (PEP) with bNAbs could clear infection and prevent reservoir establishment by treating infants with the bNAbs VRC07-523 and PGT121 beginning 24 h after viral exposure (84). This study was followed by another in infants that showed treatment at 30 h also cleared infection, while treatment at 48 h reduced or cleared viremia in only half of the animals (85). Further delaying therapy, a similarly designed study in which bNAbs 10–1,074 and 3BNC117 were given to adult macaques beginning on day 3 after SHIV exposure had a progressively worse outcome, with the majority of animals becoming productively infected and, in about half of cases, eventually controlling viremia with T cell responses (86). In contrast, treatment with bNAbs PGT121 and VRC07-523 initiated on day 10, followed by 13 weeks of ART, was ineffective for clearing SHIV infection and did not alter the time to rebound or level of viral control after ART interruption (ATI), although CD4+ T cell-associated viral DNA was somewhat reduced (87). Together, these findings suggest that the window of opportunity for effective PEP to clear infection with bNAbs delivered by passive transfer is limited to the earliest days after exposure. Efficacy declines precipitously with the rapid seeding of the persistent reservoir during early acute infection.

**Table 1:** Protective efficacy of bNAbs delivered as PEP in infant and adult macaques.

**NHP model**	**bNAb**	**Target**	**Dose**	**Treatment day post-challenge**	**Outcome**	**References**
SHIV_SF162P3_, intravaginal (adults)	PGT121	V3-glycan	2 mg/kg (1×)	−1	12/12 (100%) protected from durable infection; tissue virus detected at day 1–3 in in three NHPs was cleared by days 7–10	(75)
SHIV_SF162P3_, oral (infants)	PGT121 and VRC07-523	V3 glycan and CD4bs	5 mg/kg each (4×) 40 mg/kg total	1, 4, 7, 10	4/4 (100%) aviremic for 6 months	(84)
			20 mg/kg each (4×) 160 mg/kg total	1, 4, 7, 10	6/6 (100%) aviremic for 6 months (with serial necropsy of additional NHPs showing tissue virus detected at day 2 was cleared by day 7–14)	
SHIV_SF162P3_, oral (infants)	PGT121 and VRC07-523	V3 glycan and CD4bs	20 mg/kg each (1×) 40 mg/kg total	1.25	6/6 (100) aviremic for 8 month	(85)
			5 mg/kg each (4×) 40 mg/kg total	2, 4, 7, 10	3/6 (50%) aviremic or transient blips over 10 months	
SHIV_AD8EO_, intrarectal, or intravenous (adults)	10–1,074 and 3BNC117	V3 glycan and CD4bs	10 mg/kg each (3×) 60 mg/kg total	3, 10, 17	3/13 (23%) aviremic or transient blips for 12 months	(86)
SHIV_SF162P3_, intravenous (adults)	PGT121 and VRC07-523	V3 glycan and CD4bs	20 mg/kg each (1×) 40 mg/kg total	10 (followed by ART on days 21–112)	0/6 (0%) aviremic–all rebounded after ART	(87)

If bNAbs are maintained at a therapeutic concentration by expressing them *in vivo*, animals are fully resistant to infection, and in the setting of chronic infection, able to maintain viremic suppression (88). This approach has been accomplished in both mice (89) and in NHPs using AAV as a vector to deliver modified bNAbs for expression and maintenance. Proof of principle was first shown by expressing an SIV NAb in macaques that provided resistance to challenge with SIV (90). Continued work to refine this approach led to the successful delivery and sustained production of several bNAbs that could suppress viremia in established infection, leading to prolonged control in the “Miami monkey” (91), although three of four animals had strong anti-drug antibody (ADA) responses to all AAV-delivered bNAbs that quickly eliminated their expression. These encouraging data suggest that it might be possible to use vector delivery for antibody expression in the setting of established infection and this delivery approach is discussed more below.

### Progressing Toward bNAb Prophylaxis and Therapy in the Clinic

Clinical trials to test HIVIG with neutralizing activity for efficacy in treating or blocking infection with HIV were stymied not only by the variability of different HIVIG products, but also by the difficulty in identifying subjects in the earliest stages of infection. Efforts in the 1990s to treat those with advanced infection in a double blinded trial showed modest effects on opportunistic infections, but the data only showed trends toward benefit (92). Nonetheless, these studies showed that infusion of HIVIG was not a cause of disease enhancement, which had been a concern prior to this trial. Investigators also attempted to show whether HIVIG might show the benefit of reduced vertical HIV transmission when combined with Zidovudine (AZT) in pregnant mothers and their babies in the Pediatric AIDS Clinical Trial Protocol 185 in Uganda (93). This trial resulted in an unexpectedly low transmission rate overall, and the effects of the addition of HIVIG could not be assessed.

The success with bNAbs in NHP models and the added potency and breadth of recently characterized bNAbs has engendered significant enthusiasm to test them in clinical trials in established infection and as both PEP and PrEP. Studies in NHPs described in the previous section suggest that the antiviral effects provided by bNAbs would last only as long as they are maintained above the therapeutic level unique to each bNAb-virus pairing, and thus repeated administrations will be necessary to maintain this level if they are used when infection is well-established. One of the attractive concepts put forth in recent years is that passive treatment with bNAbs might afford a “drug holiday” during treatment interruption if they could be shown to maintain plasma viremia at undetectable levels for significant time periods.

A *sine qua non* for clinical studies is safety, and there is a strong and growing compendium of data to show bNAbs are well-tolerated at a range of doses in early-stage safety trials (39, 94). Based on this emerging safety profile and the promising data from animal models, paired clinical trials, designated the antibody mediate prophylaxis (AMP) study (HVTN 704/HPTN 085), were performed to test moderately potent bNAb VRC01 for its ability to block HIV infection as PrEP in adult men and women (95). This was a large, double-blinded study that took place in the US, Europe, and Africa, and it accrued a number of positive outcomes, despite the failure to show efficacy against HIV acquisition overall. First, the trial participation was excellent and repeated (10) infusions every 4 weeks of the antibody preparation at 10 or 30 mg/kg were well-tolerated in all sites. By studying the transmitted viruses, it was possible to discern whether VRC01 could prevent the transmission of HIV strains that were sensitive to this antibody. The data showed that the incidence of infection with VRC01-sensitive isolates (IC_80_ <1 ug per milliliter, as measured by the laboratory assay used) per 100 person-years was 0.20 among VRC01 recipients and 0.86 among placebo recipients, which translates to an estimated prevention efficacy of 75.4% (95% Confidence Interval, 45.5–88.9). Thus, this trial showed for the first time that a single bNAb can block HIV infection in humans, if the virus is sensitive to that bNAb, and represents a landmark logistical achievement. High adherence rates were observed in both study arms, with all doses delivered averaging to 9 per recipient (out of 10 intended) and an average loss of follow-up of only 7.85% per year. Importantly, the trial also validated the use of an *in vitro* assay that uses a pseudovirus readout, the TZM-bl assay. This finding means that it should now be possible to more accurately predict which antibodies, or antibody cocktails, can be used and at what dose, to gain coverage for different parts of the world. Future trials will be needed to determine whether this approach is feasible for broader application.

The use of bNAbs as a PEP therapy for HIV has been explored as well, with mixed results. The first test of viral suppression in very early (acute) and established infection utilized a cocktail of first generation bNAbs given at the time of ART interruption (96). Importantly, the study showed a delay in viral rebound in the early infection group, compared with acutely infected controls, and it also showed that *in vivo* effects were due primarily to the bNAb 2G12. A trial of VRC01 in ART-untreated people with chronic HIV achieved only transient decreases in viremia (97). Consistent with NHP studies, the use of more than one bNAb was deemed likely to be required to prevent escape. To test cocktails of potent bNAbs, Michel Nussenzweig and colleagues enrolled HIV-infected subjects with well-controlled viremia under ART and tested a cocktail of bNAbs prior to ATI, followed by monitoring for continued viral suppression. Importantly, the viruses in these individuals were pre-screened for sensitivity to the passive bNAbs planned for the administration during ATI. There were several encouraging results from this study. This cocktail consisted of bNAbs 10–1,074 and 3BNC117 and resulted in more substantial reductions in viremia for a longer period, with no evidence of viral resistance evolving to either antibody (98). In ART-treated participants undergoing experimental treatment interruption (ATI), VRC01 delayed viral rebound only slightly compared with historical controls and exerted selective pressure on the rebounding viral variants, highlighting the risk of monotherapy for viral resistance (99). Again, combination therapy with 10–1,074 and 3BNC117 lengthened the duration of viral suppression after ATI to a median of 21 weeks, with no evidence of viral resistance to both bNAbs (100). Notably, the bNAb therapy enhanced the development of T cell responses to HIV, although their contribution to viral control or clinical outcomes was unclear (101). Taken together, these findings show that using potent bNAbs in combinations of two or more will maximize therapeutic efficacy and reduce the risk of viral escape, particularly when bNAbs like 1–18 with a low propensity for escape are included (41). If it is possible to enhance antibody activity, delivery, and persistence, the door to sustained viremic control could be opened.

### Further Considerations for Moving to the Clinic: Fc Modifications, Isotype, and Multi-Epitope Targeting

#### Fc Modifications

As bNAbs move toward widespread clinical use, numerous efforts have been made to further improve their efficacy. Modifications to reduce immunogenicity, prolong Ab half-life, and enhance effector functions have met with mixed success and continue to be intensely researched. A critical advancement has been the introduction of the M428L/N434S (LS) into the CH3 domain, which enhances affinity to the neonatal FcR (FcRn) at low endosomal pH (<6.5) and thus drives recycling of internalized antibody to the extracellular milieu rather than proceeding to proteolytic degradation (102, 103). This mutation is particularly notable for both its clinical importance and value in animal models, as it is not linked to higher levels of NHP ADA responses seen with some FcRn enhancement variants (104). Further extension of antibody half-life has recently been achieved by a complementary FcRn independent mechanism caused by the deletion of E294. This single amino acid deletion results in hypersialylation of N297, and while the exact mechanism responsible for extended half-life remains unclear, it is hypothesized that sialic acid on glycosylated N297 prevents recognition by asialoglycoprotein receptor and thus reduces antibody degradation in the liver (105). While extending half-life, the E294 deletion concurrently reduced binding to FcγRIII and complement dependent lysis of infected Raji cells (105), thus its clinical advancement may be limited to contexts where eliminating these effector functions is deemed less important than maximizing half-life.

While neutralization is the most important antibody mediated function for protection to HIV in animal models, several loss-of-function studies show that effector functions contribute, particularly as neutralization titers wane (Table 2) (78, 80, 106–109). The first report of the importance of Fc functions on bNAbs to HIV was found with moderately potent b12 against a single high dose vaginal challenge with SHIV_SF162P3_ in NHPs. Delivery of 25 mg/kg b12 1 day prior to challenge protected eight of nine (89%) macaques from infection. In contrast, pre-treatment with b12 variant L228A/L229A (LALA), with mutations that severely reduce affinity to FcγRs and largely abrogate both complement and FcγR mediated effector functions, only protected five of nine (56%) NHPs (78). A follow up study with repeated low dose challenges found similar results, where the risk of infection was reduced by a factor of 21 in b12 treated animals compared to controls but only by a factor of 10 in b12 LALA treated macaques (106).

**Table 2:** Summary of studies using HIV-1 bNAbs modified for altered Fc mediated effector functions.

**bNAb**	**Target**	**Modification**	***In vitro* effect**	**Model**	**Approach**	**Central findings**	**References**
b12	CD4bs	• LALA • KA	• FcgR and C' knockdown • C' knockdown	NHP	I.V. Ab delivery prior to single high dose SHIV_SF162p3_ mucosal challenge	Impeding Fc Receptor binding is detrimental to protection efficacy	(78)
b12	CD4bs	LALA	FcgR and C' knockdown	NHP	Low-dose repeated I.V. Ab delivery prior to initial challenge and concurrent with repeated low dose SHIV_SF162p3_ mucosal challenges	Fewer challenges were required to infect macaques compared to WT b12-treated macaques; Lower serum titers of NAb than previously tested can provide benefit against low-dose repeated challenges, and effector function may contribute to protection	(106)
3BNC117	CD4bs	GRLR	FcgR knockout	Mouse	Ab inhibition of pseudovirus dissemination in luciferase reporter mice	ADCC improves protection	(107)
3BNC117 + 10-1074 + PG16	CD4bs + V3 glycan + V2 apex	GASDALIE (all)	Increased ADCC/ADCP		Combination therapy given to HIV_YU2_ –infected humanized mice	Increased ADCC/ADCP activity results in a more pronounced and sustained reduction in viral load	
VRC07-523	CD4bs	LALA	FcgR and C' null	NHP	I.V. Ab treatment of NHPs chronically infected with SHIV_SF162p3_	Effector function was responsible for 21% of the decline in plasma viral load	(108)
		DEL	Increased FcgR binding, ADCC; C' knockdown			Enhanced FcgR3 binding induced NK cell necroptosis and abrogated the 21% decline in viral load	
117/1400	CD4bs/V2 apex	TM and NA	FcgR knockout	Mouse & NHP	I.P. Ab treatment of HIV-1_JR−CSF_ (mouse) or SHIV_BG505_ (NHP) chronically infected animals	Effector function was responsible for 25–45% (mouse) or 31% (NHP) of the decline in plasma viral load	(109)
N6-LS	CD4bs	GASDALIE	Increased ADCC/ADCP	Mouse	I.P. Ab treatment of HIV-1_JR−CSF_ chronically infected mice	Enhanced effector function marginally increases viral decay	
		TM and NA	FcgR knockout	Mouse	I.P. Ab treatment of HIV-1_pNL(AD8)_ chronically infected mice	Effector function was responsible for 39% of the decline in plasma viral load	
PGT121	V3 glycan	LALA	FcgR and C' knockdown	NHP	I.V. Ab delivery prior to single SHIV_SF162p3_ I.V. challenge	Complete protection in both groups (1 mg/kg)	(110)
					NK depletion then I.V. Ab delivery prior to challenge with cell associated SHIV_SF162p3_	Complete protection in both groups (1 mg/kg)	
					I.V. Ab treatment of NHPs chronically infected with SHIV_SF162p3_	No difference in plasma viral decay, cell associated virus, or time to rebound	
PGT121	V3 glycan	LALA	FcgR and C' knockdown	NHP	I.V. Ab delivery prior to single SHIV_SF162p3_ vaginal challenge	Equivalent protection in both groups (0.2 mg/kg)	(111)
		LALAPG	FcgR ablation and C' knockdown	NHP	I.V. Ab delivery prior to single SHIV_SF162p3_ vaginal challenge	Equivalent protection in both groups (0.2 mg/kg)	

Observations from these original studies were expanded in both humanized mice and NHP models. Bournazos and colleagues showed that mice pretreated intraperitoneally with a sub-protective dose of 3BNC117 subtype IgG2a, which binds mouse activating but not inhibitory FcRs with substantially greater affinity than IgG1, displayed significantly lower viral RNA copies following HIV pseudovirus challenge than those treated with IgG1 subtype, and this discrepancy was abrogated in *Fc*γ*R*^−/−−^ mice (107). Additional experiments with a luciferase reporter mice model showed that low doses (20 μg) of 3BNC117 modified for increased activating FcγR engagement [G236A/S239D/A330L/I332E (GASDALIE)] significantly lowered infection compared to unmodified 3BNC117. Consistently, delivery of an FcγR null binding variant [G236R/L328R (GRLR)] showed greater infection than with unmodified Ab. This observation was repeated, but with a smaller discrepancy, using a higher dose of 50 μg 3BNC117. Finally, this study examined Fc-enhanced combination therapy with GASDALIE or GRLR modified 3BNC117, 10–1,074, and PG16 therapeutically administered to HIV_YU−2_ infected humanized mice and saw a more pronounced and sustained reduction in viral load in the GASDALIE group, with only 33% of mice viremic compared to 90% in the GRLR group 6 weeks after sustained Ab treatment.

It should be noted that in the bNAb cocktail experiment neutralizing activity was the most important determinant of protection and Fc-dependent differences were only observed at midrange titers (only from 10 μg dose among a 0.1–100 μg dose range). Additionally, pseudovirus experiments may not necessarily forecast the ability of Ab mediated effector function to limit viral dissemination and reservoir establishment with replication competent T/F viruses. Nonetheless, this groundbreaking work in humanized mice highlighted the contribution of native effector functions of several bNAbs and suggested the potential for increasing their antiviral contributions through enhancing affinity to FcγR.

It has since been shown that in numerous additional contexts, Fc mediated effector functions aid reducing blood viremia following treatment of established infections. Wang and colleagues showed that engineered bi-specific bNAb 117/1,400 targeting the CD4bs and the V2 apex (from parent bNAbs 3BNC117 and PGDM1400) caused a 25–45% greater reduction in the plasma viral load of humanized mice infected with HIV JR-CSF than the Fc null 117/1,400 variant (109). Similarly, in NHPs infected with SHIV BG505 the plasma virus decay slope in 117/1400 treated macaques was 31% greater than the Fc null variant, with a mirroring result in PBMC associated SHIV RNA. A third experiment in humanized mice infected with HIV pNL(AD8) and bNAb N6-LS found a greater 39% reduction in plasma viral load than in the Fc-Null group (109). Thus, Fc mediated effector functions contribute substantially (21–45%) to virus control in many treatment settings.

These conclusions, however, do not extend to all bNAbs, as seen when the V3-glycan supersite targeting bnAbs PGT121 and PGT121 LALA were tested both prophylactically and therapeutically in pigtail macaques (110). The sterilizing immunity observed in both groups pretreated with Ab followed by SHIV_SF162p3_ intravenous challenge suggest both groups maintained neutralization titers high enough to make Fc function irrelevant until virus was cleared, and this conclusion is reinforced by the similar result obtained following ~40–80% depletion of NK cells in macaques prior to challenge. To gain further insight, chronically infected macaques were treated with PGT121 or PGT121 LALA, but no difference was observed in plasma viral decay rates, time to rebound, or PBMC-associated viral DNA. The decay results seem to be at odds with those seen with b12; however, it is possible that Fc contribution is most relevant in a protection setting where neutralization, while remaining the principal factor, is insufficient to protect. Examining PGT121 similarly delivered followed by a repeated low dose challenge would be of interest to ascertain if PGT121 Fc function does indeed tilt the scale toward prolonged protection as neutralization activity wanes.

Confoundingly, while *Parsons et al*. did show FcγRIIIa binding (ELISA), NK cell activation (flow for CD107a^+^), and ADCC activity (flow infected cell elimination) occurred with PGT121, but not PGT121 LALA, it has elsewhere been reported that PGT121 and fellow glycan-supersite targeting bnAbs 10–1,074 and PGT151 bind B and T cells of HIV infected and uninfected individuals without distinction (111). This non-specific binding is mediated by complex glycans and can result in the NK cell activation irrespective of the presence of HIV antigen. These observations, together with the discrepancy between antiviral effector function contributions attributed to PGT121 *in vitro* and found for other bNAbs *in vivo*, warranted further investigation.

A robust follow up study was performed by Hangartner et al. and their results further validated the finding that effector functions on PGT121 are dispensable for combating infection (112). Through a series of experiments in rhesus macaques, the Hangartner study methodically tested and observed protection–first, at the same dose of 1 mg/kg with PGT121 and PGT121 LALA, and then bNAb doses were reduced by 5-fold to 0.2 mg/kg. Partial protection was seen at this lower dose in both groups with 7 of 15 protected by PGT121 and 11 of 15 protected by PGT121 LALA, a result that showed no significant difference between the groups. In another follow-up experiment, PGT121 LALA, that was determined to have some residual FcγR1 binding, was replaced with PGT121 LALALPG to ensure ablation of all binding to macaque FcγRs. Remarkably, using the same single high-dose challenge and the lower sub-protective dose of 0.2 mg/kg, PGT121-LALAPG protected 12 of 15 animals. Notably, this study had several key elements that were different from that performed by Parsons et al. (110) including the animal model (rhesus vs. pigtail macaques), the route of infection (vaginal vs. intravenous challenge), virus preparation (cell-free vs. cell-based), timing of bNAb delivery before challenge (1 day vs. 1 h) and yet, the final conclusion in the two studies was identical. Together, these studies strongly support the conclusion that under the conditions tested, effector function does not contribute to PGT121 protection.

An interesting observation from the Hangartner study is the possibility of a slower rate of diffusion into tissue by PGT121 LALA, although the calculated half-life was comparable to that of unmodified PGT121. Investigations of biodistribution with Fc-modified bNAbs may reveal new evidence that could influence therapeutic applications. The authors propose several reasonable theories to explain the contradicting outcomes with PGT121 compared to the original study of FcR importance (78). The two explanations that seem most likely are (1) the mechanism of protection provided by the bNAbs currently in play may differ in their inherent use or requirement for engaging FcγRs *in vivo*. For example, the Env epitope targeted could hinder engagement of some subclasses and facilitate others; neutralization potency may supersede the need for effector function; or the inherent effector function profile of a particular isotype may dictate efficacy. Re-engineered bNAbs or isotype switching could alter FcγR interactions leading to modified effector functions that may or may not augment protection by neutralization. (2) The wide diversity of assays, techniques, and cell lines employed by multiple laboratories can lend expectations based on *in vitro* results that do not adequately represent the dynamics of *in vivo* antibody function. Taken together, these studies underscore that neutralization is the proven correlate of protection that will largely inform bNAb selection for clinical applications.

Nevertheless, the Fc functional knockout studies reviewed here and indirect evidence reviewed elsewhere (113) highlight the importance of engaging effector cells in the context of HIV, and efforts have been made to improve the contributions of effector functions in clinically relevant settings. Numerous Fc mutations have been identified that enhance binding to FcγRs and/or complement components *in vitro* (114–120). Separate groups using different bNAb/HIV pairings have shown the GASDALIE variant, which is selectively focused on increasing ADCC and ADCP activity, exhibits greater viral control than unmodified bNAb in humanized mice (107, 109). However, these results have not been reproducible in NHPs. Asokan and colleagues performed a robust study where rhesus macaques infected with SHIV_SF162P3_ were treated with CD4bs targeting bNAb VRC07-523-LS either Fc unmodified, with a the S329D/A330L/I332E (DEL) mutation that selectively enhances engagement to FcγRs (particularly FcγRIII), or with the complement/FcγR null variant LALA. Similar to the results by Wang and colleagues reviewed above, animals treated with unmodified VRC07-523-LS had a 21% greater decline in plasma viral load than that of the Fc null group. Unexpectedly, the DEL variant induced necroptosis on FcγR bearing cells, leading to the pronounced depletion of NK cells, monocytes, and mDCs from the blood over the first day post treatment and yielding an Fc functional null phenotype with blood viremia matching that of the LALA group (108).

Overall, these studies show a substantial anti-viral contribution of effector functions mediated by many bNAbs to HIV (21–45%) with the remaining activity attributed to neutralization. They additionally underscore both the importance of animal models and the need to be mindful of species-specific differences between humanized mice, NHPs, and humans when evaluating effector functions, including distinct expression patterns of FcγRs on host cells and antibody or receptor glycosylation profiles (113, 121, 122). It remains unclear whether native Fc mediated effector functions can be improved for clinical use using alternative variants to focus the engagement of FcγRs or complement, a question to which ongoing research by ourselves and others will provide more clarity.

#### Isotype and Multi-Epitope Targeting

For bNAbs to achieve their full public health potential, the modalities of isotype and cocktail design should be further considered. While most therapeutic antibodies are delivered as IgG1 to preserve maximal half-life and retain FcγR binding, other isotypes may be warranted in specific situations. For example, increased phagocytosis and improved complement activation has been seen *in vitro* with IgG3 mAbs (123), but to date, our group has conducted the sole *in vivo* passive transfer with a human HIV-targeting IgG3 Ab in non-human primates. The anti-V2 mAb we used is not a bNAb, meagerly neutralizes the SHIV challenge virus, and only modestly mediates ADCC. Consequently, IgG1 or IgG3 mAb 830A provided no advantage in protection against SHIV_SF162P3_ in macaques (124). The prospect of subclass switching to convert bNAbs into IgG3 antibodies is gaining some traction as another approach to improve Ab-driven effective humoral immune responses, and further exploration with IgG3 in a preclinical setting has been suggested (125) especially where effector functions could complement or augment neutralization.

Starting ART after sustained chronic infection leaves HIV+ individuals with immense genetic diversity within viral reservoirs and impedes efficacy of bNAb therapy by presenting greater pre-existing resistance. Wilson and Lynch address this conundrum in an excellent review (126) describing approaches that could keep strategies for bNAb therapy afloat. For example, patients could be screened to select optimal bNAbs for therapy against circulating species, optimizing chances for success. This approach for more individualized treatment in future applications is a logical choice for treatment such as targeting host proteins or engineered multi-targeting antibodies become more available. The requirement for cocktails of bNAbs has long been realized, but new ideas for combination therapy include using specifically engineered antibody molecules with the capability to target multiple epitopes (127–130), such as bi-specific antibodies (bsAbs) (131–133), with the 10E8.4/iMab now in clinical trials. Bispecific T-cell engagers (BiTEs) have been developed so far primarily for oncology (134–136), but now also for HIV therapeutics (137). Also, Dual-Affinity Re-Targeting protein (DARTs) (138–140), have been tested in preclinical animal models and some have moved into phase 1 clinical trials. Whether the therapy consists of mAbs alone or augmented with engineered features that generate superhero antibodies, the key component of any approach will require the combination of targeting different specificities and having molecules with complementary functions.

Critical to the future of measuring the success of antibody-based therapy will be the impact of all strategies on viral reservoirs present during ART suppression. Also needed are further investigations of the prevalence of tissue-bound virus during bNAb or ART suppression. Pre-clinical studies in non-human primates are beginning to inform on bNAb biodistribution and targeting foci of infection (141). Directing bNAbs to sites of HIV cellular reservoirs has been proposed under the premise that they could be used in concert with or following latency reversal agents (LRA) and then target the reactivated virus and infected cells presenting Env. The neutralization potency against free virus combined with bNAb Fc interacting with FcγRs would be a strong force to eliminate infected cells via innate effector cells, such as natural killer cells, monocytes, and neutrophils (142). Studying 10 individuals with extended periods of viral suppression, Tuyishime et al. reported that combinations of neutralizing and non-neutralizing mAbs were able to mediate ADCC and eliminate cells infected with viruses that were resistant to neutralization by the tested mAbs (143). The group found that a combination of 3 mAbs worked best for NK-specific killing viruses that were resistant to individual or multiple bNAbs. It is still too early to know how effectively bNAbs will contribute to an eventual HIV cure strategy, and it is likely that advancing Fab and Fc engineering tactics will be required for success.

Even with bNAb cocktails and multi-epitope targeting as strategies for impeding HIV, it is important to note that several studies have shown higher concentrations of antibody are required to neutralize virus in the context of cell-associated transmission of HIV (144, 145) and reviewed in (146). When considering treatment or prophylaxis applications for HIV infection, diminished inhibition of cell-to-cell spread could represent a critical chink in the armor of bNAbs. Li et al. investigated this phenomenon using a novel CD4+ T cell assay to assess transmission from infected to uninfected cells *via* virological synapses (147). A greater amount of antibody was required to inhibit infection against two T/F HIV isolates (QH0692, RHPA) and two lab-adapted clones (JR-FL, NL4-3) for all bNAbs tested: (i) glycan patch-recognizing antibody 2G12; (ii) CD4bs antibodies VRC01 and HJ16; (iii) MPER antibodies 2F5 and 4E10; (iv) gp120/gp41 interface antibody 35O22; (v) V1/V2 apex antibody PG9; and (vi) glycan-dependent V3 antibodies PGT121, PGT126, and 10–1,074. For all four virus strains tested, cell-to-cell infection was generally more resistant to CD4bs VRC01 compared to HJ16. When compared, MPER bNAbs 2F5 and 4E10 showed similar decrease in potency and maximum efficacy with some differences depending upon which virus was tested. Surprisingly, neutralization potency was most detrimentally affected in cell-to-cell infection with the gp120/gp41 interface bNAb 35,022. V1V2 targeting bNAb PG9 does not potently neutralize either of the viruses tested, and not surprisingly, failed to inhibit cell-free or cell-to-cell transmission. As expected with the glycan-dependent and potent neutralizing bNAbs PGT121, PGT126, and 10–1,074, almost complete inhibition was seen in cell-free infection of all T/F viruses, but these bNAbs also required substantially greater amounts of antibody to prevent cell-to-cell transmission.

For bNAbs that target glycan-dependent and glycan-related epitopes, neutralization plateaus below 100% (148). In the Li experiments, the glycan-associated bNAbs showed a drop in inhibition from 95 to 36%. In an earlier study, the group has also reported an effect by the YXXL motif in the CT of gp41 that could allosterically modulate Env on the surface of the infected cell that could impact sensitivity to bNAbs (149). A full explanation of the notable and possibly consequential decrease in virus inhibition in cell-to-cell infection is still unclear and warrants further investigations, especially as bNAbs are advanced in the clinic.

### Clinical Delivery in Pursuit of a Functional Cure

Current clinically approved mAbs are delivered by passive transfer either by intravenous infusion or subcutaneous injection. While bNAbs to HIV are still in development, it is notable that other IgG preparations or mAbs are licensed for several other severe or lethal virus infections including hepatitis B [treatment of infants with hepatitis B immune globulin (HBIG) during vaccination], respiratory syncytial virus, Ebola, and SARS-CoV-2, and there is preexisting infrastructure to manufacture mAbs at scale (150–153). Newly discovered mAbs for treatment of infectious diseases and cancer are typically produced in mammalian cell lines and optimally yield 3–8 g/L (154), and efforts continue to be made to improve production efficiency (155). Alternative plant-based production approaches have also been implemented for the production of mAbs for prevention and treatment, and these retain their anti-viral functional profile despite the variance in post translational modifications (156–158).

Two alternative approaches to bNAb delivery are being investigated that, if successfully advanced, have the potential to maintain bNAbs at therapeutic levels for very long periods of time and approach a functional cure. One personalized approach is to collect memory B cells from an individual living with HIV and replace the BCR with a bNAb of choice through CRISPR/Cas9 editing, then infuse the engineered cells back into the individual, mirroring the approach used for CAR-T cell therapy approved for some lymphomas (159, 160). This approach has shown success in mouse models performed by different groups introducing bNAb VRC01 (161) or 3BNC117 (162) as the BCR, with *in vivo* transfer resulting in a memory B cell population that retains the ability to class switch, undergo somatic hypermutation, and clonally expand and differentiate into plasmablasts following immunization with HIV Env.

Vectoring genes for *in vivo* expression of HIV-neutralizing antibodies opens the door to an alternate pathway to a functional cure. Adenovirus-associated virus (AAV) is non-pathogenic, widely distributed in the human population, and exists as multiple serotypes. As mentioned earlier, the first demonstration of *in vivo* expression of anti-SIV Env antibodies in rhesus macaques occurred more than a decade ago and demonstrated the effectiveness of AAV to infect and transduce macaques (163). The concept of using AAV for delivery of SIV neutralizing antibody genes in macaques was tested later and resulted in long-lived neutralizing activity and full protection from SIV infection (90). These studies were followed by transferring multiple genes expressing HIV antibodies into SHIV-infected macaques using a modified AAV vector that resulted in remarkable viral control. By concept, co-expressing multiple antibodies in the study evaluated the effect of bNAb cocktails on SHIV infection (91). While AAV delivery of bNAb genes has been fairly successful in macaques, there are some important issues with this approach that currently limit development in humans, including: (1) variability in antibody expression; (2) failure to fully clear the viral reservoir; (3) and generation of anti-drug antibodies (ADA) leading to the rapid elimination of the bNAbs (164).

## Conclusion

The extended progression of bNAb therapeutic development can be envisioned as the continual nurturing of ideas and technology toward the goal of maturing to clinical efficacy. As summarized here, there has been spectacular progress in the field of antibodies as drugs to treat HIV, both as prophylaxis and as therapy. We have illustrated the cultivation and nurturing of bNAb discoveries as a tree growing from seeds to full maturity (Figure 1). Beginning from the early experiments with polyclonal Ig from humans and chimpanzees in the 1980s through the next three decades to the present status of advancement into clinical settings, we envisioned the progress in the context of organic growth. Following bNAb discoveries, several critical infusions of technology and the expansion and access to large cohorts of subjects have shaped and fostered bNAb clinical applications. Along the way, the discoveries advanced by the HIV research community and pressure to advance technology for the sole purpose of mining human immune repertoires for powerful HIV NAbs has also impacted Ab based research for other emerging pathogens. Most notably, the extraordinary speed to clone and produce clinical grade NmAbs against SARS-CoV-2 is largely due to the technology developed in advancing HIV bNAbs. In addition, bNAb lineage identification and the corresponding discovery of vulnerable Env epitopes are driving rational immunogen design aimed at coaching bNAb development through vaccination (165–167). While still in early stages, this approach has shown comparative success over other vaccines in preclinical studies and has moved to early clinical trials (168).

Even now, HIV bNAbs cloned from B cells from a rare minority of chronically infected individuals are continuing to provide clues for longer lasting and less toxic alternatives to the current standard of care. Advances in high throughput single cell characterization with BCR sequence coupled to functional binding data prior to cloning will continue to drive the discovery of novel bNAbs and expand selection for therapeutic combinations. Studies in humanized mice and non-human primates have demonstrated bNAb efficacy for both treatment and prevention when maintained at therapeutic levels defined by the individual bNAb-virus combinations. Early clinical trials using bNAb combinations show a strong safety profile, and a recently completed efficacy trial has provided proof-of-principle that bNAbs can act as PrEP against bNAb-sensitive viral strains, as well as established *in vitro* functional metrics that will inform future large scale efficacy trials in humans. As importantly, this trial demonstrated the feasibility of repeated intravenous administration with strong adherence to the infrequent dosing regimen among diverse populations, and it provides a framework for the logistical adjustments needed for continued global use. Fc engineering efforts have been successful at extending bNAb half-lives and elucidating the role of effector functions for several bNAbs, although it is unclear whether native Fc contributions can be improved. Additionally, novel modes of delivery through adoptive transfer of bNAb expressing B cells or vectored delivery represent theoretically viable ways to maintain therapeutic levels over extended periods of time and are being investigated as approaches toward a functional cure.

## Author Contributions

All authors contributed ideas and writing to this review article. The figures were developed by AH with input from NH and DS.

## Conflict of Interest

The authors declare that the research was conducted in the absence of any commercial or financial relationships that could be construed as a potential conflict of interest.

## References

[1] United Nations. UNAIDS Data. Geneva: United Nations (2019).

[2] Siliciano JD Siliciano RF. Nonsuppressible HIV-1 viremia: a reflection of how the reservoir persists. J Clin Invest. (2020) 130:5665–7. 10.1172/JCI141497 33016925 PMC7598039

[3] Swindells S Andrade-Villanueva JF Richmond GJ Rizzardini G Baumgarten A Masia M. Long-Acting cabotegravir and rilpivirine for maintenance of HIV-1 suppression. N Engl J Med. (2020) 382:1112–23. 10.1056/NEJMoa1904398 32130809

[4] Orkin C Arasteh K Gorgolas Hernandez-Mora M Pokrovsky V Overton ET Girard PM. Long-Acting cabotegravir and rilpivirine after oral induction for HIV-1 infection. N Engl J Med. (2020) 382:1124–35. 10.1056/NEJMoa1909512 32130806

[5] Stolbach A Paziana K Heverling H Pham P. A review of the toxicity of HIV medications II: interactions with drugs and complementary and alternative medicine products. J Med Toxicol. (2015) 11:326–41. 10.1007/s13181-015-0465-0 26036354 PMC4547966

[6] Margolis AM Heverling H Pham PA Stolbach A. A review of the toxicity of HIV medications. J Med Toxicol. (2014) 10:26–39. 10.1007/s13181-013-0325-8 23963694 PMC3951641

[7] Holmes D. FDA paves the way for pre-exposure HIV prophylaxis. Lancet. (2012) 380:325. 10.1016/S0140-6736(12)61235-5 22852138

[8] Emil von Behring. Facts Nobel Prize.org. Nobel Media AB (2021).

[9] Deeks SG Walker BD. Human immunodeficiency virus controllers: mechanisms of durable virus control in the absence of antiretroviral therapy. Immunity. (2007) 27:406–16. 10.1016/j.immuni.2007.08.010 17892849

[10] Bailey JR Williams TM Siliciano RF Blankson JN. Maintenance of viral suppression in HIV-1-infected HLA-B^*^57+ elite suppressors despite CTL escape mutations. J Exp Med. (2006) 203:1357–69. 10.1084/jem.20052319 16682496 PMC2121215

[11] Eichberg JW Murthy KK Ward RH Prince AM. Prevention of HIV infection by passive immunization with HIVIG or CD4-IgG. AIDS Res Hum Retrovir. (1992) 8:1515. 10.1089/aid.1992.8.1515 1466993

[12] Levy J Youvan T Lee ML. Passive hyperimmune plasma therapy in the treatment of acquired immunodeficiency syndrome: results of a 12-month multicenter double-blind controlled trial. The passive hyperimmune therapy study group. Blood. (1994) 84:2130–5. 10.1182/blood.V84.7.2130.bloodjournal8472130 7919326

[13] Vittecoq D Chevret S Morand-Joubert L Heshmati F Audat F Bary M. Passive immunotherapy in AIDS: a double-blind randomized study based on transfusions of plasma rich in anti-human immunodeficiency virus 1 antibodies vs. transfusions of seronegative plasma. Proc Natl Acad Sci USA. (1995) 92:1195–9. 10.1073/pnas.92.4.1195 7862660 PMC42665

[14] Morand-Joubert L Vittecoq D Roudot-Thoraval F Mariotti M Lefrere F Heshmati F. Virological and immunological data of AIDS patients treated by passive immunotherapy (transfusions of plasma rich in HIV-1 antibodies). Vox Sang. (1997) 73:149–54. 10.1046/j.1423-0410.1997.7330149.x 9358616

[15] Zhu P Liu J Bess J Chertova E Lifson JD Grisé H. Distribution and three-dimensional structure of AIDS virus envelope spikes. Nature. (2006) 441:847–52. 10.1038/nature04817 16728975

[16] Behrens AJ Vasiljevic S Pritchard LK Harvey DJ Andev RS Krumm SA. Composition and antigenic effects of individual glycan sites of a trimeric HIV-1 envelope glycoprotein. Cell Rep. (2016) 14:2695–706. 10.1016/j.celrep.2016.02.058 26972002 PMC4805854

[17] Cao L Diedrich JK Kulp DW Pauthner M He L Park SR. Global site-specific N-glycosylation analysis of HIV envelope glycoprotein. Nat Commun. (2017) 8:14954. 10.1038/ncomms14954 28348411 PMC5379070

[18] Silver ZA Antonopoulos A Haslam SM Dell A Dickinson GM Seaman MS. Discovery of O-linked carbohydrate on HIV-1 envelope and its role in shielding against one category of broadly neutralizing antibodies. Cell Rep. (2020) 30:1862–9.e4. 10.1016/j.celrep.2020.01.056 32049016 PMC7904042

[19] Lu M Ma X Castillo-Menendez LR Gorman J Alsahafi N Ermel U. Associating HIV-1 envelope glycoprotein structures with states on the virus observed by smFRET. Nature. (2019) 568:415–9. 10.1038/s41586-019-1101-y 30971821 PMC6655592

[20] Alsahafi N Bakouche N Kazemi M Richard J Ding S Bhattacharyya S. An Asymmetric opening of HIV-1 envelope mediates antibody-dependent cellular cytotoxicity. Cell Host Microbe. (2019) 25:578–87. 10.1016/j.chom.2019.03.002 30974085 PMC6592637

[21] Kwong PD Mascola JR. HIV-1 vaccines based on antibody identification, B cell ontogeny, epitope structure. Immunity. (2018) 48:855–71. 10.1016/j.immuni.2018.04.029 29768174

[22] Liao HX Lynch R Zhou T Gao F Alam SM Boyd SD. Co-evolution of a broadly neutralizing HIV-1 antibody and founder virus. Nature. (2013) 496:469–76. 10.1038/nature12053 23552890 PMC3637846

[23] Bonsignori M Kreider EF Fera D Meyerhoff RR Bradley T Wiehe K. Staged induction of HIV-1 glycan-dependent broadly neutralizing antibodies. Sci Transl Med. (2017) 9:eaai7514. 10.1126/scitranslmed.aai7514 28298420 PMC5562350

[24] de Bree GJ Wheatley AK Lynch RM Prabhakaran M Grijsen ML Prins JM. Longitudinal dynamics of the HIV-specific B cell response during intermittent treatment of primary HIV infection. PLoS ONE. (2017) 12:e0173577. 10.1371/journal.pone.0173577 28296911 PMC5351995

[25] Sok D Burton DR. Recent progress in broadly neutralizing antibodies to HIV. Nat Immunol. (2018) 19:1179–88. 10.1038/s41590-018-0235-7 30333615 PMC6440471

[26] Safrit JT Fung MS Andrews CA Braun DG Sun WN Chang TW. hu-PBL-SCID mice can be protected from HIV-1 infection by passive transfer of monoclonal antibody to the principal neutralizing determinant of envelope gp120. AIDS. (1993) 7:15–21. 10.1097/00002030-199301000-00002 7680205

[27] Okamoto Y Eda Y Ogura A Shibata S Amagai T Katsura Y. In SCID-hu mice, passive transfer of a humanized antibody prevents infection and atrophic change of medulla in human thymic implant due to intravenous inoculation of primary HIV-1 isolate. J Immunol. (1998) 160:69–76. 9551957

[28] Klein F Halper-Stromberg A Horwitz JA Gruell H Scheid JF Bournazos S. HIV therapy by a combination of broadly neutralizing antibodies in humanized mice. Nature. (2012) 492:118–22. 10.1038/nature11604 23103874 PMC3809838

[29] Ruprecht RM Ferrantelli F Kitabwalla M Xu W McClure HM. Antibody protection: passive immunization of neonates against oral AIDS virus challenge. Vaccine. (2003) 21:3370–3. 10.1016/S0264-410X(03)00335-9 12850342

[30] Baba TW Liska V Hofmann-Lehmann R Vlasak J Xu W Ayehunie S. Human neutralizing monoclonal antibodies of the IgG1 subtype protect against mucosal simian-human immunodeficiency virus infection. Nat Med. (2000) 6:200–6. 10.1038/72309 10655110

[31] Hessell AJ Rakasz EG Poignard P Hangartner L Landucci G Forthal DN. Broadly neutralizing human anti-HIV antibody 2G12 is effective in protection against mucosal SHIV challenge even at low serum neutralizing titers. PLoS Pathog. (2009) 5:e1000433. 10.1371/journal.ppat.1000433 19436712 PMC2674935

[32] Parren PW Marx PA Hessell AJ Luckay A Harouse J Cheng-Mayer C. Antibody protects macaques against vaginal challenge with a pathogenic R5 simian/human immunodeficiency virus at serum levels giving complete neutralization in vitro. J Virol. (2001) 75:8340–7. 10.1128/JVI.75.17.8340-8347.2001 11483779 PMC115078

[33] Hessell AJ Rakasz EG Tehrani DM Huber M Weisgrau KL Landucci G. Broadly neutralizing monoclonal antibodies 2F5 and 4E10 directed against the human immunodeficiency virus type 1 gp41 membrane-proximal external region protect against mucosal challenge by simian-human immunodeficiency virus SHIVBa-L. J Virol. (2010) 84:1302–13. 10.1128/JVI.01272-09 19906907 PMC2812338

[34] Gautam R Nishimura Y Pegu A Nason MC Klein F Gazumyan A. A single injection of anti-HIV-1 antibodies protects against repeated SHIV challenges. Nature. (2016) 533:105–9. 10.1038/nature17677 27120156 PMC5127204

[35] Julg B Liu PT Wagh K Fischer WM Abbink P Mercado NB. Protection against a mixed SHIV challenge by a broadly neutralizing antibody cocktail. Sci Transl Med. (2017) 9:eaao4235. 10.1126/scitranslmed.aao4235 28931655 PMC5747528

[36] Julg B Sok D Schmidt SD Abbink P Newman RM Broge T. Protective efficacy of broadly neutralizing antibodies with incomplete neutralization activity against SHIV in rhesus monkeys. J Virol. (2017) 91:e01187–17. 10.1128/JVI.01187-17 28768869 PMC5625479

[37] Julg B Tartaglia LJ Keele BF Wagh K Pegu A Sok D. Broadly neutralizing antibodies targeting the HIV-1 envelope V2 apex confer protection against a clade C SHIV challenge. Sci Transl Med. (2017) 9:eaal1321. 10.1126/scitranslmed.aal1321 28878010 PMC5755978

[38] NIH. National Institute of Allergy and Infectious Diseases, Evaluating the Safety, Tolerability, Pharmacokinetics, and Antiviral Activity of Combinations of Monoclonal Antibodies PGT121, PGDM1400, 10-1074, and VRC07-523LS Administered Via Intravenous Infusion in Healthy, HIV-uninfected Adult Participants. ClinicalTrials.gov NCT03928821. Bethesda: NIH (2019).

[39] Cohen YZ Butler AL Millard K Witmer-Pack M Levin R Unson-O'Brien C. Safety, pharmacokinetics, and immunogenicity of the combination of the broadly neutralizing anti-HIV-1 antibodies 3BNC117 and 10-1074 in healthy adults: a randomized, phase 1 study. PLoS ONE. (2019) 14:e0219142. 10.1371/journal.pone.0219142 31393868 PMC6687118

[40] Crowell TA Colby DJ Pinyakorn S Sacdalan C Pagliuzza A Intasan J. Safety and efficacy of VRC01 broadly neutralising antibodies in adults with acutely treated HIV (RV397): a phase 2, randomised, double-blind, placebo-controlled trial. Lancet HIV. (2019) 6:e297–306. 10.1016/S2352-3018(19)30053-0 31000477 PMC6693657

[41] Schommers P Gruell H Abernathy ME Tran MK Dingens AS Gristick HB. Restriction of HIV-1 escape by a highly broad and potent neutralizing antibody. Cell. (2020) 180:471–89. 10.1016/j.cell.2020.01.010 32004464 PMC7042716

[42] Shen CH DeKosky BJ Guo Y Xu K Gu Y Kilam D. VRC34-Antibody lineage development reveals how a required rare mutation shapes the maturation of a broad HIV-neutralizing lineage. Cell Host Microbe. (2020) 27:531–43.e6. 10.1016/j.chom.2020.01.027 32130953 PMC7467872

[43] Zhang L Irimia A He L Landais E Rantalainen K Leaman DP. An MPER antibody neutralizes HIV-1 using germline features shared among donors. Nat Commun. (2019) 10:5389–404. 10.1038/s41467-019-12973-1 31772165 PMC6879610

[44] Williams LD Ofek G Schatzle S McDaniel JR Lu X Nicely NI. Potent and broad HIV-neutralizing antibodies in memory B cells and plasma. Sci Immunol. (2017) 2:eaa12200. 10.1126/sciimmunol.aal2200 28783671 PMC5905719

[45] Williams WB Zhang J Jiang C Nicely NI Fera D Luo K. Initiation of HIV neutralizing B cell lineages with sequential envelope immunizations. Nat Commun. (2017) 8:1732. 10.1038/s41467-017-01336-3 29170366 PMC5701043

[46] Haynes BF Fleming J St Clair EW Katinger H Stiegler G Kunert R. Cardiolipin polyspecific autoreactivity in two broadly neutralizing HIV-1 antibodies. Science. (2005) 308:1906–8. 10.1126/science.1111781 15860590

[47] Haynes BF Moody MA Verkoczy L Kelsoe G Alam SM. Antibody polyspecificity and neutralization of HIV-1: a hypothesis. Hum Antibodies. (2005) 14:59–67. 10.3233/HAB-2005-143-402 16720975 PMC2673565

[48] Lee JH Ozorowski G Ward AB. Cryo-EM structure of a native, fully glycosylated, cleaved HIV-1 envelope trimer. Science. (2016) 351:1043–8. 10.1126/science.aad2450 26941313 PMC5001164

[49] Setliff I Shiakolas AR Pilewski KA Murji AA Mapengo RE Janowska K. High-Throughput mapping of b cell receptor sequences to antigen specificity. Cell. (2019) 179:1636–46. 10.1016/j.cell.2019.11.003 31787378 PMC7158953

[50] Zost SJ Gilchuk P Chen RE Case JB Reidy JX Trivette A. Rapid isolation and profiling of a diverse panel of human monoclonal antibodies targeting the SARS-CoV-2 spike protein. Nat Med. (2020) 26:1422–7 10.1038/s41591-020-0998-x 32651581 PMC8194108

[51] Zost SJ Gilchuk P Case JB Binshtein E Chen RE Nkolola JP. Potently neutralizing and protective human antibodies against SARS-CoV-2. Nature. (2020) 584:443–9. 10.1038/s41586-020-2548-6 32668443 PMC7584396

[52] Bangaru S Lang S Schotsaert M Vanderven HA Zhu X Kose N. A site of vulnerability on the influenza virus hemagglutinin head domain trimer interface. Cell. (2019) 177:1136–52.e18. 10.1016/j.cell.2019.04.011 31100268 PMC6629437

[53] Gilchuk P Kuzmina N Ilinykh PA Huang K Gunn BM Bryan A. Multifunctional Pan-ebolavirus antibody recognizes a site of broad vulnerability on the ebolavirus glycoprotein. Immunity. (2018) 49:363–74.e10. 10.1016/j.immuni.2018.06.018 30029854 PMC6104738

[54] Vogt MR Fu J Kose N Williamson LE Bombardi R Setliff I. Human antibodies neutralize enterovirus D68 and protect against infection and paralytic disease. Sci Immunol. (2020) 5:eaba4902. 10.1126/sciimmunol.aba4902 32620559 PMC7418079

[55] Keele BF Jones JH Terio KA Estes JD Rudicell RS Wilson ML. Increased mortality and AIDS-like immunopathology in wild chimpanzees infected with SIVcpz. Nature. (2009) 460:515–9. 10.1038/nature08200 19626114 PMC2872475

[56] Novembre GJ Saucier M Anderson DC Klumpp SA O'Neil SP Brown CRI. Development of AIDS in a chimpanzee infected with human immunodeficiency virus type 1. J Virol. (1997) 71:4086–91. 10.1128/JVI.71.5.4086-4091.1997 9094687 PMC191562

[57] Shultz LD Ishikawa F Greiner DL. Humanized mice in translational biomedical research. Nat Rev Immunol. (2007) 7:118–30. 10.1038/nri2017 17259968

[58] Marsden MD. Benefits and limitations of humanized mice in HIV persistence studies. Retrovirology. (2020) 17:7. 10.1186/s12977-020-00516-2 32252791 PMC7137310

[59] Deruaz M Moldt B Le KM Power KA Vrbanac VD Tanno S. Protection of humanized mice from repeated intravaginal HIV challenge by passive immunization: a model for studying the efficacy of neutralizing antibodies *in vivo*. J Infect Dis. (2016) 214:612–6. 10.1093/infdis/jiw203 27357340 PMC4957443

[60] Balazs AB Ouyang Y Hong CM Chen J Nguyen SM Rao DS. Vectored immunoprophylaxis protects humanized mice from mucosal HIV transmission. Nat Med. (2014) 20:296–300. 10.1038/nm.3471 24509526 PMC3990417

[61] Fultz PN. Nonhuman primate models for AIDS. Clin Infect Dis. (1993) 17 (Suppl. 1):S230–5. 10.1093/clinids/17.Supplement_1.S230 8399921

[62] Li J Lord CI Haseltine W Letvin NL Sodroski J. Infection of cynomolgus monkeys with a chimeric HIV-1/SIVmac virus that expresses the HIV-1 envelope glycoproteins. J Acquir Immune Defic Syndr. (1988) 5:639–46. 1613662

[63] Harouse JM Gettie A Tan RC Blanchard J Cheng-Mayer C. Distinct pathogenic sequela in rhesus macaques infected with CCR5 or CXCR4 utilizing SHIVs. Science. (1999) 284:816–9. 10.1126/science.284.5415.816 10221916

[64] Humes D Emery S Laws E Overbaugh J. A species-specific amino acid difference in the macaque CD4 receptor restricts replication by global circulating HIV-1 variants representing viruses from recent infection. J Virol. (2012) 86:12472–83. 10.1128/JVI.02176-12 22973036 PMC3497638

[65] Li H Wang S Lee FH Roark RS Murphy AI Smith J. New SHIVs and improved design strategy for modeling HIV-1 transmission, immunopathogenesis, prevention and cure. J Virol. (2021) 71:21. 10.1128/JVI.00071-21 33658341 PMC8139694

[66] Bauer AM Bar KJ. Advances in simian–human immunodeficiency viruses for nonhuman primate studies of HIV prevention and cure. Curr Opin HIV AIDS. (2020) 15:275–81. 10.1097/COH.0000000000000645 32769631

[67] Nelson AN Goswami R Dennis M Tu J Mangan RJ Saha PT. SHIV.CH505-infected infant and adult rhesus macaques exhibit similar HIV Env-specific antibody kinetics, despite distinct T-follicular helper (Tfh) and germinal center B cell landscapes. J Virol. (2019) 93:15. 10.1128/JVI.00168-19 31092583 PMC6639294

[68] Bar KJ Coronado E Hensley-McBain T O'Connor MA Osborn JM Miller C. Simian-Human immunodeficiency virus SHIV.CH505 infection of rhesus macaques results in persistent viral replication and induces intestinal immunopathology. J Virol. (2019) 93:e00372–19. 10.1128/JVI.00372-19 31217249 PMC6714786

[69] Hessell AJ Haigwood NL. Animal models in HIV-1 protection and therapy. Curr Opin HIV AIDS. (2015) 10:170–6. 10.1097/COH.0000000000000152 25730345 PMC4526147

[70] Pegu A Hessell AJ Mascola JR Haigwood NL. Use of broadly neutralizing antibodies for HIV-1 prevention. Immunol Rev. (2017) 275:296–312. 10.1111/imr.12511 28133803 PMC5314445

[71] Berendam SJ Nelson AN Goswami R Persaud D Haigwood NL Chahroudi A. Pediatric HIV: the potential of immune therapeutics to achieve viral remission and functional cure. Curr HIV/AIDS Rep. (2020) 17:237–48. 10.1007/s11904-020-00495-1 32356090 PMC7296986

[72] Shibata R Igarashi T Haigwood N Buckler-White A Ogert R Ross W. Neutralizing antibody directed against the HIV-1 envelope glycoprotein can completely block HIV-1/SIV chimeric virus infections of macaque monkeys. Nature Med. (1999) 5:204–10. 10.1038/5568 9930869

[73] Igarashi T Brown C Azadegan A Haigwood N Dimitrov D Martin MA. Human immunodeficiency virus type 1 neutralizing antibodies accelerate clearance of cell-free virions from blood plasma. Nat Med. (1999) 5:211–6. 10.1038/5576 9930870

[74] Shingai M Donau OK Plishka RJ Buckler-White A Mascola JR Nabel GJ. Passive transfer of modest titers of potent and broadly neutralizing anti-HIV monoclonal antibodies block SHIV infection in macaques. J Exp Med. (2014) 211:2061–74. 10.1084/jem.20132494 25155019 PMC4172223

[75] Liu J Ghneim K Sok D Bosche WJ Li Y Chipriano E. Antibody-mediated protection against SHIV challenge includes systemic clearance of distal virus. Science. (2016) 353:1045–49. 10.1126/science.aag0491 27540005 PMC5237379

[76] Julg B Pegu A Abbink P Liu J Brinkman A Molloy K. Virological control by the CD4-binding site antibody N6 in SHIV-infected rhesus monkeys. J Virol. (2017) 91:e00498–17. 10.1128/JVI.00498-17 28539448 PMC5533891

[77] Pegu A Borate B Huang Y Pauthner MG Hessell AJ Julg B. A meta-analysis of passive immunization studies shows that serum-neutralizing antibody titer associates with protection against SHIV challenge. Cell Host Microbe. (2019) 26:336–46.e3. 10.1016/j.chom.2019.08.014 31513771 PMC6755677

[78] Hessell AJ Hangartner L Hunter M Havenith CE Beurskens FJ Bakker JM. Fc receptor but not complement binding is important in antibody protection against HIV. Nature. (2007) 449:101–4. 10.1038/nature06106 17805298

[79] Burton DR Hessell AJ Keele BF Klasse PJ Ketas TA Moldt B. Limited or no protection by weakly or nonneutralizing antibodies against vaginal SHIV challenge of macaques compared with a strongly neutralizing antibody. Proc Natl Acad Sci USA. (2011) 108:11181–6. 10.1073/pnas.1103012108 21690411 PMC3131343

[80] Astronomo RD Santra S Ballweber-Fleming L Westerberg KG Mach L Hensley-McBain T. Neutralization takes precedence over IgG or IgA isotype-related functions in mucosal HIV-1 antibody-mediated protection. EBioMedicine. (2016) 14:97–111. 10.1016/j.ebiom.2016.11.024 27919754 PMC5161443

[81] Cheeseman HM Olejniczak NJ Rogers PM Evans AB King DF Ziprin P. Broadly neutralizing antibodies display potential for prevention of HIV-1 infection of mucosal tissue superior to that of nonneutralizing antibodies. J Virol. (2017) 91:e01762–16. 10.1128/JVI.01762-16 27795431 PMC5165208

[82] Rudicell RS Kwon YD Ko SY Pegu A Louder MK Georgiev IS . Enhanced potency of a broadly neutralizing HIV-1 antibody *in vitro* improves protection against lentiviral infection in vivo. J Virol. (2014) 88:12669–82. 10.1128/JVI.02213-14 25142607 PMC4248941

[83] Haigwood NL Montefiori DC Sutton WF McClure J Watson AJ Voss G. Passive immunotherapy in simian immunodeficiency virus-infected macaques accelerates the development of neutralizing antibodies. J Virol. (2004) 78:5983–95. 10.1128/JVI.78.11.5983-5995.2004 15140996 PMC415787

[84] Hessell AJ Jaworski JP Epson E Matsuda K Pandey S Kahl C. Early short-term treatment with neutralizing human monoclonal antibodies halts SHIV infection in infant macaques. Nat Med. (2016) 22:362–8. 10.1038/nm.4063 26998834 PMC4983100

[85] Shapiro MB Cheever T Malherbe DC Pandey S Reed J Yang ES. Single-dose bNAb cocktail or abbreviated ART post-exposure regimens achieve tight SHIV control without adaptive immunity. Nat Commun. (2020) 11:70. 10.1038/s41467-019-13972-y 31911610 PMC6946664

[86] Nishimura Y Gautam R Chun TW Sadjadpour R Foulds KE Shingai M. Early antibody therapy can induce long-lasting immunity to SHIV. Nature. (2017) 543:559–563. 10.1038/nature21435 28289286 PMC5458531

[87] Bolton DL Pegu A Wang K McGinnis K Nason M Foulds K. Human Immunodeficiency virus type 1 monoclonal antibodies suppress acute simian-human immunodeficiency virus viremia and limit seeding of cell-associated viral reservoirs. J Virol. (2015) 90:1321–32. 10.1128/JVI.02454-15 26581981 PMC4719604

[88] Grobben M Stuart RA van Gils MJ. The potential of engineered antibodies for HIV-1 therapy and cure. Curr Opin Virol. (2019) 38:70–80. 10.1016/j.coviro.2019.07.007 31421319

[89] van den Berg FT Makoah NA Ali SA Scott TA Mapengo RE Mutsvunguma LZ. AAV-Mediated expression of broadly neutralizing and vaccine-like antibodies targeting the HIV-1 envelope V2 region. Mol Ther Methods Clin Dev. (2019) 14:100–12. 10.1016/j.omtm.2019.06.002 31334303 PMC6616373

[90] Johnson PR Schnepp BC Zhang J Connell MJ Greene SM Yuste E. Vector-mediated gene transfer engenders long-lived neutralizing activity and protection against SIV infection in monkeys. Nat Med. (2009) 15:901–6. 10.1038/nm.1967 19448633 PMC2723177

[91] Martinez-Navio JM Fuchs SP Pantry SN Lauer WA Duggan NN Keele BF. Adeno-Associated virus delivery of Anti-HIV monoclonal antibodies can drive long-term virologic suppression. Immunity. (2019) 50:567–75.e5. 10.1016/j.immuni.2019.02.005 30850342 PMC6457122

[92] Jacobson JM Colman N Ostrow NA Simson RW Tomesch D Marlin L. Passive immunotherapy in the treatment of advanced human immunodeficiency virus infection. J Infect Dis. (1993) 168:298–305. 10.1093/infdis/168.2.298 8101550

[93] Stiehm ER Lambert JS Mofenson LM Bethel J Whitehouse J Nugent R., Glenn Fowler M, Mathieson BJ, Reichelderfer P, Nemo GJ, Korelitz J, Meyer WA III, et al. Efficacy of zidovudine and human immunodeficiency virus (HIV) hyperimmune immunoglobulin for reducing perinatal HIV transmission from HIV-infected women with advanced disease: results of pediatric AIDS clinical trials group protocol 185. J Infect Dis. (1999) 179:567–75. 10.1086/314637 9952362

[94] Ledgerwood JE Coates EE Yamshchikov G Saunders JG Holman L Enama ME. Safety, pharmacokinetics, and neutralization of the broadly neutralizing HIV-1 human monoclonal antibody VRC01 in healthy adults. Clin Exp Immunol. (2015) 182:289–301. 10.1111/cei.12692 26332605 PMC4636891

[95] Corey L Gilbert PB Juraska M Montefiori DC Morris L Karuna ST. Two randomized trials of neutralizing antibodies to prevent HIV-1 acquisition. N Engl J Med. (2021) 384:1003–14. 10.1056/NEJMoa2031738 33730454 PMC8189692

[96] Trkola A Kuster H Rusert P Joos B Fischer M Leemann C. Delay of HIV-1 rebound after cessation of antiretroviral therapy through passive transfer of human neutralizing antibodies. Nat Med. (2005) 11:615–22. 10.1038/nm1244 15880120

[97] Lynch RM Boritz E Coates EE DeZure A Madden P Costner P. Virologic effects of broadly neutralizing antibody VRC01 administration during chronic HIV-1 infection. Sci Transl Med. (2015) 7:319ra206. 10.1126/scitranslmed.aad5752 26702094 PMC12366723

[98] Bar-On Y Gruell H Schoofs T Pai JA Nogueira L Butler AL. Safety and antiviral activity of combination HIV-1 broadly neutralizing antibodies in viremic individuals. Nat Med. (2018) 24:1701–1707. 10.1038/s41591-018-0186-4 30258217 PMC6221973

[99] Bar KJ Sneller MC Harrison LJ Justement JS Overton ET Petrone ME. Effect of HIV antibody VRC01 on viral rebound after treatment interruption. N Engl J Med. (2016) 375:2037–50. 10.1056/NEJMoa1608243 27959728 PMC5292134

[100] Mendoza P Gruell H Nogueira L Pai JA Butler AL Millard K. Combination therapy with anti-HIV-1 antibodies maintains viral suppression. Nature. (2018) 561:479–84. 10.1038/s41586-018-0531-2 30258136 PMC6166473

[101] Niessl J Baxter AE Mendoza P Jankovic M Cohen YZ Butler AL. Combination anti-HIV-1 antibody therapy is associated with increased virus-specific T cell immunity. Nat Med. (2020) 26:222–7. 10.1038/s41591-019-0747-1 32015556 PMC7018622

[102] Zalevsky J Chamberlain AK Horton HM Karki S Leung IW Sproule TJ. Enhanced antibody half-life improves in vivo activity. Nat Biotechnol. (2010) 28:157–9. 10.1038/nbt.1601 20081867 PMC2855492

[103] Ko SY Pegu A Rudicell RS Yang ZY Joyce MG Chen X. Enhanced neonatal Fc receptor function improves protection against primate SHIV infection. Nature. (2014) 514:642–5. 10.1038/nature13612 25119033 PMC4433741

[104] Rosenberg YJ Lewis GK Montefiori DC LaBranche CC Lewis MG Urban LA. Introduction of the YTE mutation into the non-immunogenic HIV bnAb PGT121 induces anti-drug antibodies in macaques. PLoS ONE. (2019) 14:e0212649. 10.1371/journal.pone.0212649 30785963 PMC6437720

[105] Bas M Terrier A Jacque E Dehenne A Pochet-Béghin V Beghin C. Fc sialylation prolongs serum half-life of therapeutic antibodies. J Immunol. (2019) 202:1582–94. 10.4049/jimmunol.1800896 30683704

[106] Hessell AJ Poignard P Hunter M Hangartner L Tehrani DM Bleeker WK. Effective, low-titer antibody protection against low-dose repeated mucosal SHIV challenge in macaques. Nat Med. (2009) 15:951–4. 10.1038/nm.1974 19525965 PMC4334439

[107] Bournazos S Klein F Pietzsch J Seaman MS Nussenzweig MC Ravetch JV. Broadly neutralizing anti-HIV-1 antibodies require Fc effector functions for in vivo activity. Cell. (2014) 158:1243–53. 10.1016/j.cell.2014.08.023 25215485 PMC4167398

[108] Asokan M Dias J Liu C Maximova A Ernste K Pegu A. Fc-mediated effector function contributes to the in vivo antiviral effect of an HIV neutralizing antibody. Proc Natl Acad Sci. USA. (2020) 117:18754–63. 10.1073/pnas.2008236117 32690707 PMC7414046

[109] Wang P Gajjar MR Yu J Padte NN Gettie A Blanchard JL. Quantifying the contribution of Fc-mediated effector functions to the antiviral activity of anti-HIV-1 IgG1 antibodies *in vivo*. Proc Natl Acad Sci USA. (2020) 117:18002–9. 10.1073/pnas.2008190117 32665438 PMC7395461

[110] Parsons MS Lee WS Kristensen AB Amarasena T Khoury G Wheatley AK. Fc-dependent functions are redundant to efficacy of anti-HIV antibody PGT121 in macaques. J Clin Invest. (2019) 129:182–91. 10.1172/JCI122466 30475230 PMC6307963

[111] Blazkova J Refsland EW Clarridge KE Shi V Justement JS Huiting ED. Glycan-dependent HIV-specific neutralizing antibodies bind to cells of uninfected individuals. J Clin Invest. (2019) 29:4832–7. 10.1172/JCI125955 31589168 PMC6819136

[112] Hangartner L Beauparlant D Rakasz E Nedellec R Hoze N McKenney K. Effector function does not contribute to protection from virus challenge by a highly potent HIV broadly neutralizing antibody in nonhuman primates. Sci Transl Med. (2021) 13:eabe3349. 10.1126/scitranslmed.abe3349 33731434 PMC8049513

[113] Crowley AR Ackerman ME. Mind the gap: how interspecies variability in IgG and its receptors may complicate comparisons of human and non-human primate effector function. Front Immunol. (2019) 10:697–716. 10.3389/fimmu.2019.00697 31024542 PMC6463756

[114] Dufloo J Guivel-Benhassine F Buchrieser J Lorin V Grzelak L Dupouy E. Anti-HIV-1 antibodies trigger non-lytic complement deposition on infected cells. EMBO Rep. (2020) 21:e49351. 10.15252/embr.201949351 31833228 PMC10563447

[115] Moore GL Chen H Karki S Lazar GA. Engineered Fc variant antibodies with enhanced ability to recruit complement and mediate effector functions. MAbs. (2010) 2:181–9. 10.4161/mabs.2.2.11158 20150767 PMC2840237

[116] Lazar GA Dang W Karki S Vafa O Peng JS Hyun L. Engineered antibody Fc variants with enhanced effector function. Proc Natl Acad Sci USA. (2006) 103:4005–10. 10.1073/pnas.0508123103 16537476 PMC1389705

[117] Diebolder CA Beurskens FJ de Jong RN Koning RI Strumane K Lindorfer MA. Complement is activated by IgG hexamers assembled at the cell surface. Science. (2014) 343:1260–3. 10.1126/science.1248943 24626930 PMC4250092

[118] Oostindie SC van der Horst HJ Lindorfer MA Cook EM Tupitza JC Zent CS. CD20 and CD37 antibodies synergize to activate complement by Fc-mediated clustering. Haematologica. (2019) 104:1841–52. 10.3324/haematol.2018.207266 30792198 PMC6717598

[119] de Jong RN Beurskens FJ Verploegen S Strumane K van Kampen MD Voorhorst M. A novel platform for the potentiation of therapeutic antibodies based on antigen-dependent formation of IgG hexamers at the cell surface. PLoS Biol. (2016) 14:e1002344. 10.1371/journal.pbio.1002344 26736041 PMC4703389

[120] Moldt B Schultz N Dunlop DC Alpert MD Harvey JD Evans DT. A panel of IgG1 b12 variants with selectively diminished or enhanced affinity for Fcgamma receptors to define the role of effector functions in protection against HIV. J Virol. (2011) 85:10572–81. 10.1128/JVI.05541-11 21849450 PMC3187489

[121] Boesch AW Miles AR Chan YN Osei-Owusu NY Ackerman ME. IgG Fc variant cross-reactivity between human and rhesus macaque FcgammaRs. MAbs. (2017) 9:455–65. 10.1080/19420862.2016.1274845 28055295 PMC5384711

[122] Chan YN Boesch AW Osei-Owusu NY Emileh A Crowley AR Cocklin SL. IgG binding characteristics of rhesus macaque FcgammaR. J Immunol. (2016) 197:2936–47. 10.4049/jimmunol.1502252 27559046 PMC5026948

[123] Chu TH Crowley AR Backes I Chang C Tay M Broge T. Hinge length contributes to the phagocytic activity of HIV-specific IgG1 and IgG3 antibodies. PLoS Pathog. (2020) 16:e1008083. 10.1371/journal.ppat.1008083 32092122 PMC7058349

[124] Hessell AJ Shapiro MB Powell R Malherbe DC McBurney SP Pandey S. Reduced cell-associated DNA and improved viral control in macaques following passive transfer of a single anti-V2 monoclonal antibody and repeated SHIV challenges. J Virol. (2018) 92:e02198–17. 10.1128/JVI.02198-17 29514914 PMC5952134

[125] Chu TH Patz EF Jr Ackerman ME. Coming together at the hinges: therapeutic prospects of IgG3. MAbs. (2021) 13:1882028. 10.1080/19420862.2021.1882028 33602056 PMC7899677

[126] Wilson A Lynch RM. Embracing diversity: how can broadly neutralizing antibodies effectively target a diverse HIV-1 reservoir? Curr Opin Pharmacol. (2020) 54:173–8. 10.1016/j.coph.2020.10.004 33189993

[127] Xu L Pegu A Rao E Doria-Rose N Beninga J McKee K. Trispecific broadly neutralizing HIV antibodies mediate potent SHIV protection in macaques. Science. (2017) 358:85–90. 10.1126/science.aan8630 28931639 PMC5978417

[128] Steinhardt JJ Guenaga J Turner HL McKee K Louder MK O'Dell S. Rational design of a trispecific antibody targeting the HIV-1 Env with elevated anti-viral activity. Nat Commun. (2018) 9:877. 10.1038/s41467-018-03335-4 29491415 PMC5830440

[129] Khan SN Sok D Tran K Movsesyan A Dubrovskaya V Burton DR. Targeting the HIV-1 spike and coreceptor with Bi- and trispecific antibodies for single-component broad inhibition of entry. J Virol. (2018) 92:e00384–18. 10.1128/JVI.00384-18 29976677 PMC6146690

[130] Padte NN Yu J Huang Y Ho DD. Engineering multi-specific antibodies against HIV-1. Retrovirology. (2018) 15:60. 10.1186/s12977-018-0439-9 30157871 PMC6114543

[131] Asokan M Rudicell RS Louder M McKee K O'Dell S Stewart-Jones G. Bispecific antibodies targeting different epitopes on the HIV-1 envelope exhibit broad and potent neutralization. J Virol. (2015) 89:12501–12. 10.1128/JVI.02097-15 26446600 PMC4665248

[132] Bournazos S Gazumyan A Seaman MS Nussenzweig MC Ravetch JV. Bispecific Anti-HIV-1 antibodies with enhanced breadth and potency. Cell. (2016) 165:1609–20. 10.1016/j.cell.2016.04.050 27315478 PMC4970321

[133] Fabozzi G Pegu A Koup RA Petrovas C. Bispecific antibodies: potential immunotherapies for HIV treatment. Methods. (2019) 154:118–24. 10.1016/j.ymeth.2018.10.010 30352254 PMC6348037

[134] Huang Y Yu J Lanzi A Yao X Andrews CD Tsai L. Engineered bispecific antibodies with exquisite HIV-1-neutralizing activity. Cell. (2016) 165:1621–31. 10.1016/j.cell.2016.05.024 27315479 PMC4972332

[135] Moore PA Zhang W Rainey GJ Burke S Li H Huang L. Application of dual affinity retargeting molecules to achieve optimal redirected T-cell killing of B-cell lymphoma. Blood. (2011) 117:4542–51. 10.1182/blood-2010-09-306449 21300981

[136] Nagorsen D Baeuerle PA. Immunomodulatory therapy of cancer with T cell-engaging BiTE antibody blinatumomab. Exp Cell Res. (2011) 317:1255–60. 10.1016/j.yexcr.2011.03.010 21419116

[137] Brozy J Schlaepfer E Mueller CKS Rochat MA Rampini SK Myburgh R. Antiviral activity of HIV gp120-targeting bispecific T cell engager antibody constructs. J Virol. (2018) 92:e00491–18. 10.1128/JVI.00491-18 29720517 PMC6026749

[138] Chichili GR Huang L Li H Burke S He L Tang Q. A CD3xCD123 bispecific DART for redirecting host T cells to myelogenous leukemia: preclinical activity and safety in nonhuman primates. Sci Transl Med. (2015) 7:289ra82. 10.1126/scitranslmed.aaa5693 26019218

[139] Sung JA Pickeral J Liu L Stanfield-Oakley SA Lam CY Garrido C. Dual-Affinity Re-Targeting proteins direct T cell-mediated cytolysis of latently HIV-infected cells. J Clin Invest. (2015) 125:4077–90. 10.1172/JCI82314 26413868 PMC4639974

[140] Sloan DD Lam CY Irrinki A Liu L Tsai A Pace CS. Targeting HIV reservoir in infected CD4 T cells by dual-affinity re-targeting molecules (DARTs) that bind HIV envelope and recruit cytotoxic T cells. PLoS Pathog. (2015) 11:e1005233. 10.1371/journal.ppat.1005233 26539983 PMC4634948

[141] Santangelo PJ Rogers KA Zurla C Blanchard EL Gumber S Strait K. Whole-body immunoPET reveals active SIV dynamics in viremic and antiretroviral therapy-treated macaques. Nat Methods. (2015) 12:427–32. 10.1038/nmeth.3320 25751144 PMC4425449

[142] Rossignol E Alter G Julg B. Antibodies for human immunodeficiency virus-1 cure strategies. J Infect Dis. (2021) 223:22–31. 10.1093/infdis/jiaa165 33586772 PMC7883024

[143] Tuyishime M Garrido C Jha S Moeser M Mielke D LaBranche C. Improved killing of HIV-infected cells using three neutralizing and non-neutralizing antibodies. J Clin Invest. (2020) 130:5157–70. 10.1172/JCI135557 32584790 PMC7524508

[144] Abela IA Berlinger L Schanz M Reynell L Gunthard HF Rusert P. Cell-cell transmission enables HIV-1 to evade inhibition by potent CD4bs directed antibodies. PLoS Pathog. (2012) 8:e1002634. 10.1371/journal.ppat.1002634 22496655 PMC3320602

[145] Reh L Magnus C Schanz M Weber J Uhr T Rusert P. Capacity of broadly neutralizing antibodies to inhibit HIV-1 cell-cell transmission is strain- and epitope-dependent. PLoS Pathog. (2015) 11:e1004966. 10.1371/journal.ppat.1004966 26158270 PMC4497647

[146] Malbec M Porrot F Rua R Horwitz J Klein F Halper-Stromberg A. Broadly neutralizing antibodies that inhibit HIV-1 cell to cell transmission. J Exp Med. (2013) 210:2813–21. 10.1084/jem.20131244 24277152 PMC3865481

[147] Li H Zony C Chen P Chen BK. Reduced potency and incomplete neutralization of broadly neutralizing antibodies against cell-to-cell transmission of HIV-1 with transmitted founder envs. J Virol. (2017) 91:e02425–16. 10.1128/JVI.02425-16 28148796 PMC5391450

[148] McCoy LE Falkowska E Doores KJ Le K Sok D van Gils MJ. Incomplete neutralization and deviation from sigmoidal neutralization curves for HIV broadly neutralizing monoclonal antibodies. PLoS Pathog. (2015) 11:e1005110. 10.1371/journal.ppat.1005110 26267277 PMC4534392

[149] Durham ND Yewdall AW Chen P Lee R Zony C Robinson JE. Neutralization resistance of virological synapse-mediated HIV-1 Infection is regulated by the gp41 cytoplasmic tail. J Virol. (2012) 86:7484–95. 10.1128/JVI.00230-12 22553332 PMC3416307

[150] American Academy of Pediatrics Committee on Infectious D American Academy of Pediatrics Bronchiolitis Guidelines C. Updated guidance for palivizumab prophylaxis among infants and young children at increased risk of hospitalization for respiratory syncytial virus infection. Pediatrics. (2014) 134:415–20. 10.1542/peds.2014-1665 25070315

[151] Posner J Barrington P Brier T Datta-Mannan A. Monoclonal antibodies: past, present and future. Handb Exp Pharmacol. (2019) 260:81–141. 10.1007/164_2019_323 31820172

[152] Chen P Nirula A Heller B Gottlieb RL Boscia J Morris J. SARS-CoV-2 neutralizing antibody LY-CoV555 in outpatients with covid-19. N Engl J Med. (2021) 384:229–37. 10.1056/NEJMoa2029849 33113295 PMC7646625

[153] Hou J Cui F Ding Y Dou X Duan Z Han G. Management algorithm for interrupting mother-to-child transmission of hepatitis B virus. Clin Gastroenterol Hepatol. (2019) 17:1929–36.e1. 10.1016/j.cgh.2018.10.007 30312789

[154] Li F Vijayasankaran N Shen AY Kiss R Amanullah A. Cell culture processes for monoclonal antibody production. MAbs. (2010) 2:466–79. 10.4161/mabs.2.5.12720 20622510 PMC2958569

[155] Kelley B Kiss R Laird M. A different perspective: how much innovation is really needed for monoclonal antibody production using mammalian cell technology? Adv Biochem Eng Biotechnol. (2018) 165:443–62. 10.1007/10_2018_59 29721583

[156] Rosenberg YJ Montefiori DC LaBranche CC Lewis MG Sack M Lees JP. Protection against SHIV challenge by subcutaneous administration of the plant-derived PGT121 broadly neutralizing antibody in macaques. PLoS ONE. (2016) 11:e0152760. 10.1371/journal.pone.0152760 27031108 PMC4816452

[157] Rosenberg Y Sack M Montefiori D Labranche C Lewis M Urban L. Pharmacokinetics and immunogenicity of broadly neutralizing HIV monoclonal antibodies in macaques. PLoS ONE. (2015) 10:e0120451. 10.1371/journal.pone.0120451 25807114 PMC4373669

[158] Rosenberg Y Sack M Montefiori D Forthal D Mao L Hernandez-Abanto S. Rapid high-level production of functional HIV broadly neutralizing monoclonal antibodies in transient plant expression systems. PLoS ONE. (2013) 8:e58724. 10.1371/journal.pone.0058724 23533588 PMC3606348

[159] Voss JE Gonzalez-Martin A Andrabi R Fuller RP Murrell B McCoy LE. Reprogramming the antigen specificity of B cells using genome-editing technologies. Elife. (2019) 8:e42995. 10.7554/eLife.42995 30648968 PMC6355199

[160] Luo XM Maarschalk E O'Connell RM Wang P Yang L Baltimore D. Engineering human hematopoietic stem/progenitor cells to produce a broadly neutralizing anti-HIV antibody after in vitro maturation to human B lymphocytes. Blood. (2009) 113:1422–31. 10.1182/blood-2008-09-177139 19059876

[161] Huang D Tran JT Olson A Vollbrecht T Tenuta M Guryleva MV. Vaccine elicitation of HIV broadly neutralizing antibodies from engineered B cells. Nat Commun. (2020) 11:5850. 10.1038/s41467-020-20304-y 33203876 PMC7673113

[162] Nahmad AD Raviv Y Horovitz-Fried M Sofer I Akriv T Nataf D. Engineered B cells expressing an anti-HIV antibody enable memory retention, isotype switching and clonal expansion. Nat Commun. (2020) 11:5851. 10.1038/s41467-020-19649-1 33203857 PMC7673991

[163] Johnson PR Schnepp BC Connell MJ Rohne D Robinson S Krivulka GR. Novel adeno-associated virus vector vaccine restricts replication of simian immunodeficiency virus in macaques. J Virol. (2005) 79:955–65. 10.1128/JVI.79.2.955-965.2005 15613324 PMC538580

[164] Gardner MR Fetzer I Kattenhorn LM Davis-Gardner ME Zhou AS Alfant B. Anti-drug antibody responses impair prophylaxis mediated by AAV-delivered HIV-1 broadly neutralizing antibodies. Mol Ther. (2019) 27:650–60. 10.1016/j.ymthe.2019.01.004 30704961 PMC6403482

[165] LaBranche CC Henderson R Hsu A Behrens S Chen X Zhou T. Neutralization-guided design of HIV-1 envelope trimers with high affinity for the unmutated common ancestor of CH235 lineage CD4bs broadly neutralizing antibodies. PLoS Pathog. (2019) 15:e1008026. 10.1371/journal.ppat.1008026 31527908 PMC6764681

[166] Steichen JM Lin YC Havenar-Daughton C Pecetta S Ozorowski G Willis JR. A generalized HIV vaccine design strategy for priming of broadly neutralizing antibody responses. Science. (2019) 366:6470–6483. 10.1126/science.aax4380 31672916 PMC7092357

[167] Saunders KO Wiehe K Tian M Acharya P Bradley T Alam SM. Targeted selection of HIV-specific antibody mutations by engineering B cell maturation. Science. (2019) 366:e7199. 10.1126/science.aay7199 31806786 PMC7168753

[168] Ward AB Wilson IA. Innovations in structure-based antigen design and immune monitoring for next generation vaccines. Curr Opin Immunol. (2020) 65:50–6. 10.1016/j.coi.2020.03.013 32387642 PMC7174181

